# A Novel Combination of Factors, Termed SPIE, which Promotes Dopaminergic Neuron Differentiation from Human Embryonic Stem Cells

**DOI:** 10.1371/journal.pone.0006606

**Published:** 2009-08-12

**Authors:** Tandis Vazin, Kevin G. Becker, Jia Chen, Charles E. Spivak, Carl R. Lupica, Yongqing Zhang, Lila Worden, William J. Freed

**Affiliations:** 1 Cellular Neurobiology Research Branch, Intramural Research Program (IRP), National Institute on Drug Abuse (NIDA), National Institutes of Health (NIH), Department of Health and Human Services (DHHS), Baltimore, Maryland, United States of America; 2 Gene Expression and Genomics Unit, Intramural Research Program, National Institute on Aging (NIA), Department of Health and Human Services (DHHS), Baltimore, Maryland, United States of America; 3 School of Biotechnology, Division of Gene Technology, KTH-Royal Institute of Technology, AlbaNova University Center, Stockholm, Sweden; Chiba University Center for Forensic Mental Health, Japan

## Abstract

**Background:**

Stromal-Derived Inducing Activity (SDIA) is one of the most efficient methods of generating dopaminergic (DA) neurons from embryonic stem cells (ESC). DA neuron induction can be achieved by co-culturing ESC with the mouse stromal cell lines PA6 or MS5. The molecular nature of this effect, which has been termed “SDIA” is so far unknown. Recently, we found that factors secreted by PA6 cells provided lineage-specific instructions to induce DA differentiation of human ESC (hESC).

**Methodology/Principal Findings:**

In the present study, we compared PA6 cells to various cell lines lacking the SDIA effect, and employed genome expression analysis to identify differentially-expressed signaling molecules. Among the factors highly expressed by PA6 cells, and known to be associated with CNS development, were stromal cell-derived factor 1 (SDF-1/CXCL12), pleiotrophin (PTN), insulin-like growth factor 2 (IGF2), and ephrin B1 (EFNB1). When these four factors, the combination of which was termed SPIE, were applied to hESC, they induced differentiation to TH-positive neurons *in vitro*. RT-PCR and western blot analysis confirmed the expression of midbrain specific markers, including engrailed 1, Nurr1, Pitx3, and dopamine transporter (DAT) in cultures influenced by these four molecules. Electrophysiological recordings showed that treatment of hESC with SPIE induced differentiation of neurons that were capable of generating action potentials and forming functional synaptic connections.

**Conclusions/Significance:**

The combination of SDF-1, PTN, IGF2, and EFNB1 mimics the DA phenotype-inducing property of SDIA and was sufficient to promote differentiation of hESC to functional midbrain DA neurons. These findings provide a method for differentiating hESC to form DA neurons, without a requirement for the use of animal-derived cell lines or products.

## Introduction

There is a great interest in the possibility of using human embryonic stem cells (hESC) to produce specific cell types which might be used either in cellular therapy or as *in vitro* models of human cells. Among the most interesting cell types that can be derived from hESC are DA neurons, both because of their potential use as a therapy for Parkinson's disease (PD), and as *in vitro* models for testing drugs relevant to neurodegenerative disorders, drug abuse, and addiction.

A number of groups have reported on directing hESC to differentiate into dopamine (DA) neurons [1]–[7]. The most commonly-used technique for producing DA neurons from ESC requires a co-culture step, most often using stromal cells such as the mouse PA6 cell line, but in some cases human astrocytes or other cell lines [8]–[18]. Often, patterning factors including SHH and FGF8 are employed, but these factors are effective only following an early induction step [19], [20]. A second method involves the formation of embryoid bodies (EBs), in which case internal factors, produced by hESC, are presumably responsible for the early induction phase. This approach involves a complex series of procedures including enzymatic digestion and various isolation techniques followed by SHH and FGF8 exposure [2], [5].

The biochemical nature of the initial stage of differentiation is unknown, and whether this activity is related to the SHH-FGF8 signaling system or the organizing stimulus remains to be elucidated. Upon discovery of SDIA, it was suggested that this activity accumulates on the surface of PA6 cells [8]. Other studies have suggested a role of PA6 cell-secreted factors in the DA differentiation process [21], [22]. In a recent study, we analyzed the effects of PA6 cell surface activity and secreted factors separately, and concluded that secreted factors are primarily responsible for the DA-inducing effect, whereas cell surface activity enhanced cell survival and overall neurogenesis [23].

In view of these findings, we carried out gene expression profiling of PA6 cells to identify genes coding for soluble factors with a potential role in the DA induction of hESC. In order to select the most relevant set of molecules, we conducted comparisons between the potent PA6 cell line and mouse embryonic fibroblasts (MEF), a mouse kidney cell line MM55K, and subtypes of PA6 and MS5 lines that lack DA-inducing activity.

For clarity, we will refer to the potent PA6 cell line as PA6-DA, and PA6 subtypes as PA6-X1 and PA6-X for the remainder of this paper. The transformation of the PA6-DA cells to the PA6-X cell phenotype was an unpredictable event and unrelated to the number of passages in culture. Once transformed to the PA6-X phenotype, reversion to the PA6-DA morphological phenotype did not occur. On the basis of the gene expression analysis, we selected a set of candidate genes, including SDF-1, PTN, IGF2, Insulin-like growth factor binding protein 4 (IGFBP4), and EFNB1, and examined the role of molecules encoded by these genes in DA induction of hESC *in vitro*. The four factors SDF-1, PTN, IGF2, and EFNB1 were sufficient to induce hESC to differentiate to DA neurons.

## Materials and Methods

### Cell lines

Mitotically-inactivated MEF feeder layers (Millipore, Billerica, U. S. A.) were cultured in cell culture dishes coated with 0.1% gelatin (Millipore) with high-glucose Dulbecco's Modified Eagle's Medium (DMEM) (Invitrogen, Gaithersburg, U. S. A.), supplemented with 10% fetal bovine serum (Atlanta Biologicals, Lawrenceville, U. S. A.) and 50 U/ml Penn-Strep. The MM55K mouse kidney cell line was purchased from American Type Culture Collection (ATCC, Manassas, U. S. A.) and cultured with high-glucose DMEM containing 10% fetal bovine serum and 50 U/ml Penn-Strep. The PA6 mouse stromal cell line was obtained from Riken BioResource Center Cell Bank (Tsukuba, J. P. N.). MS5 cells were kindly provided by Dr Caruz (University of Jaén, E. S. P.). The stromal cell lines were cultured in α–minimum essential medium (Invitrogen) supplemented with 10% fetal bovine serum and 50 U/ml Penn-Strep. Sub-culturing of these cell lines was accomplished by trypsinization using 0.5–1 ml 0.05% trypsin-EDTA (Invitrogen) for 2–3 min.

hESC lines used were: BG01V2, BG02, and BG03, derived by BresaGen (Athens, U. S. A.). The BG01V2 hESC line is a variant of the hESC line BG01 [24]. Mitotically-inactivated MEFs were used as feeder cells to maintain BG01V2 in an undifferentiated state. The hESC culture medium consisted of DMEM/nutrient mixture (1∶1), supplemented with 10% Knockout serum replacement, 2 mM L-glutamine, 1 mM nonessential amino acids, 4 ng/ml bFGF (Invitrogen), 50 U/ml Penn-Strep (Invitrogen), and 0.1 mM β-mercaptoethanol (Millipore). The culture media was changed daily with routine passage of hESC on fresh MEF layers carried out once a week. hESC colonies were isolated from the MEF feeder layers with 1 mg/ml collagenase type IV (Invitrogen) treatment for approximately 1 hr. Feeder-free cultures of BG01V2 were grown on human fibronectin, 20 µg/ml, (BD Biosciences, Bedford, U. S. A.) in MEF conditioned medium (MEF-CM). MEF-CM was collected daily, filtered and supplemented with an additional 16 ng/ml of bFGF before feeding hESC. MEF cells were used for 8–10 days for CM collection. CM that had been frozen at −20°C for up to one month was also used.

### Co-culture of BG01V2 with feeder cell lines

For co-culture experiments, the cell lines were grown to confluence in 6-well tissue culture plates in their respective growth media as previously described. Media was replaced with hESC differentiation medium, comprised of Glasgow Minimum Essential Medium(GMEM) supplemented with 10% knockout serum replacement, 0.1 mM nonessential amino acids (Invitrogen), 1 mM sodium pyruvate (Sigma-Aldrich, St. Louis, U. S. A.), and 0.1 mM β-mercaptoethanol (Millipore). MEFs and stromal cells were cultured in 0.1% gelatin and collagen type-I coated plates, respectively. The PA6 subtype PA6-X exhibited rapid cell division and a lack of contact inhibition of growth. Upon confluency, PA6-X feeder layers retracted and detached from the plates. To avoid detachment, cells were inactivated with 10 µg/ml mitomycin-c (Sigma) for one hr followed by three washes with fresh medium and permitted to recover overnight. BG01V2 colonies were dissociated from the MEF feeder layer by enzymatic treatment and added to the feeders at a density of 50–100 small colonies/well corresponding to 5000–10000 cells/cm^2^. The culture medium was changed on day four and every other day thereafter. The hESC were allowed to grow and differentiate on the feeder layers for 12 days.

### Immunocytochemistry

Cultures were fixed in 4% paraformaldehyde in PBS and then incubated with the primary antibodies in blocking buffer (PBS containing 5% goat serum, 2% BSA, and 0.2% Triton X-100) at room temperature for two hr.

The following primary antibodies were used: mouse anti-Oct 3/4 (1∶50; Santa Cruz Biotechnology Inc., Santa Cruz, U. S. A.), rabbit anti-TH (1∶1000; Pel-Freez), mouse anti-TH (1∶1000; Millipore), rabbit anti–β III-tubulin (1∶2000; Promega, Madison, U. S. A.), mouse anti-nestin (1∶50; R&D Systems Inc., Minneapolis, U. S. A.), rabbit anti-GFAP (1∶2000; Dako, Carpinteria, U. S. A.), mouse anti-MAP-2 (1∶500, BD Biosciences), and rabbit anti-MAP-2 (1∶1000; Millipore). Cultures were incubated with fluorescent-labeled secondary antibodies [Alexa 488 (green) or Alexa 568 (red)-labeled goat IgG; 1∶1000; Molecular Probe] in PBS with 1% BSA for 1 hr at room temperature. The cells were rinsed three times for 5 min in PBS. Negative controls included substituting the primary antibodies with non-immune mouse and rabbit IgG (1∶100; Santa Cruz) and pre-absorption of the Oct3/4 primary antibody with its antigenic peptide (0.2 mg/ml of N-terminal Oct3/4 of human origin; Santa Cruz). To ensure the specificity of the polyclonal TH antibody, a monoclonal anti-TH antibody recognizing an epitope in the N-terminus (1∶100; Sigma) was used. Cell morphology and intracellular localization were carefully examined to confirm expression of markers β-III-tubulin, MAP2, GFAP, and nestin. Images were obtained using a Carl Zeiss Axiovert 200 M microscope (Thornwood, New York, U. S. A.).

Statistical significance of the overall differences in numbers of colonies expressing various markers among the experimental groups was tested by analysis of variance (ANOVA) followed by Tukey-Kramer multiple comparisons (GraphPad Software Inc., San Diego, U. S. A.). Differences were considered significant at *p*<0.05.

### RNA extraction and expression microarrays

For total RNA extraction, about 5×10^6^ cells from each of the five cell lines were seeded onto 100 mm dishes. After 2 days, the cells were washed two times with PBS, collected by scraping, and centrifuged. RNA-STAT 60 (Tel-Test Inc., Friendswood, U. S. A.) was used to isolate the RNA following manufacturer's instructions. RNAs derived from all the feeder cell lines were reverse-transcribed, labeled, and analyzed using the Illumina microarray platform (Sentrix Mouse-8 Expression BeadChip). Arrays were processed according to the manufacturer's instructions.

### Microarray data analysis

Z-score transformation was used to compare gene expression levels between the six cell lines independent of the original hybridization intensities [25]. To obtain fold-like change in gene expression, Z-scores were converted to Z-ratios and used for statistical analysis to select differentially-expressed genes. Statistically significant differences were based on Z-ratio changes of at least 3.0 and *p*<0.05.

Functional information in relation to the gene products and gene expression patterns were obtained from the literature or from the following databases: OMIM (http://www.ncbi.nlm.nih.gov/sites/entrez?db=omim), Source (http://genome-www5.stanford.edu/cgi-bin/source/sourceBatchSearch), Cell Migration Consortium (http://cmckb.cellmigration.org), and Allen Brain Atlas (ABA) (http://www.brain-map.org).

Significantly altered genes (Z-ratio≥3.0) were categorized using the platform gene ontology FatiGO (Fast Assignment and Transference of Information using Gene Ontology, http://www.fatigo.org) with respect to gene function including biological process and molecular function [26].

### Reverse transcriptase-polymerase chain reaction (RT-PCR)

#### Mouse feeder cell lines

Complementary DNA for the polymerase chain reaction (PCR) was reverse transcribed from total RNA isolated from each feeder cell line. cDNA was synthesized from 2 µg RNA using an oligo dT primer and 1 µL MMLV (Promega, reverse transcription kit) in a 24-µl reaction according to the manufacturer's recommendations. The cycling parameters were as follows: 65°C, 2 min; 42°C, 60 min; 72°C, 10 min. 100 units of MMLV-RT was added 10 min after the reaction was brought to 42°C. The PCR reaction components for detection of the candidate genes were as follows, 1 µL cDNA in 50 µL of PCR mix containing 5× PCR buffer, 5 µL of 15 mM MgCl2, 2 µL of 10 mM deoxyribonucleoside triphosphate (dNTP), 0.5 µL each of 100 µM primers, and 5 units of GoTaq DNA polymerase (all from Promega). The thermal cycling parameters were as follows: primary denaturation, three minutes at 94°C; 35 cycles of denaturation for 1 min at 94°C; annealing for 1 min at 55°C; extension for 1 min at 72°C and final extension for 7 min at 72°C.

Equal amounts of RNA were tested in PCR reactions under the same conditions to ensure that the RNA samples were not contaminated by genomic DNA. The housekeeping gene, glyceraldehyde-3-phosphate dehydrogenase (GAPDH), was amplified as an internal control in gene expression analysis.

Primer sequences were obtained from the PrimerBank website (http://pga.mgh.harvard.edu/primerbank/) and synthesized by Invitrogen. The primer sequences can be found in Table S1.

#### Differentiated hESC

Total RNA was isolated from BG01V2-derived EBs cultured in the presence or absence of SPIE after 17 days of differentiation using RNA STAT-60 kit (Tel-test Inc). RT-PCR was performed using a One –Step RT-PCR kit (Clontech, Mountain View, U. S. A.) according to the manufacturer's instructions. DNase was added to the RNA samples before RT-PCR. For each RT-PCR reaction, 1 µl containing 500 ng of RNA was used in a 20 µl reaction volume. The cycling parameters were 50°C for 1 hr; 94°C for 5 min, then 94°C 30 sec; 65°C 30 sec; 68°C 1 min, for 35 cycles and followed by a final extension of 5 minutes at 68°C. GAPDH was used as internal control. The primer sequences are listed in Table S1.

### Functional analysis of candidate molecules

Colonies of hESC in feeder-free conditions were removed from the tissue culture plates using a sterile cell scraper and partially dissociated by gentle pipetting. The cell clusters were resuspended in hESC culture medium without bFGF and transferred to ultra low-attachment plates (Corning Incorporated) for EB formation. The medium was changed every day. After 2–4 days, the EBs were transferred to plates precoated with poly-L-ornithine (100 µg/ml, Sigma), and then laminin (20 µg/ml, Invitrogen) and cultured in hESC medium in the presence of heparin (100 µg/ml, Sigma) and the various factors. The following final concentrations of the selected growth factors were used: SDF-1 (100 ng/ml), PTN (100 ng/ml), IGF2 (100 ng/ml), IGFBP4 (500 ng/ml), and EFNB1 (200 ng/ml); all from R&D Systems. Half of the medium was replaced with fresh medium containing growth factors on day four and every two or three days after that. The cells were allowed to differentiate under these conditions for 10–14 days.

### Protein extraction and Western blot analysis

Proteins extracted from BG01V2-derived cultures treated with SPIE, and cultures grown in the absence of SPIE, were used for Western blot analysis. Briefly, cells were washed in PBS and lysed in cold preparation of modified radioimmunoprecipitation buffer (Millipore) and treated with protease inhibitors, phenylmethylsulfonyl fluoride (1 mM), leupeptin (1 µg/ml), and aprotinin (2.5 KU). The lysates were sonicated for 10 s on ice and stored at −80°C.

Twenty micrograms of protein was separated on a 4–12% Bis-Tris, or 3–8% Tris-acetate gel (Invitrogen) and transferred at 4°C to a PVDF-FL membrane overnight. After transfer, the membrane was blocked in PBS containing 5% dried milk for one hr at room temperature. The membrane was washed three times with PBST for 15 min and incubated with primary antibodies in PBST solutions containing 1% milk overnight at 4°C. The primary antibodies used were rabbit anti-MAP2 (1∶2000; Millipore), rabbit anti-Nurr1 (1∶1000; Millipore). Mouse anti-actin antibody (1∶1000; Santa Cruz) was used as an internal loading control. The membrane was washed three times with PBST for 15 min, and then incubated for 1 hr at room temperature with IRDye 700 and IRDye 800 secondary antibodies (LI-COR Biosciences, Lincoln, U. S. A.). After extensive washing, the blots were analyzed using the LI-COR Odyssey infrared imaging system (Biosciences).

### Electrophysiological recordings

The bathing solution was prepared to be similar in ionic composition and identical in osmolarity to the DMEM/F12 culture medium that the cells were grown in. It consisted of (mM): NaCl, 150; KCl, 4; MgCl2, 1; CaCl2, 1; NaH2PO4, 0.5; HEPES (hemi-sodium), 10; glucose, 18; sucrose, 24, pH 7.4, 348 mOsm. The pipette solution consisted of (mM): KCl, 140; MgCl2, 2, EGTA,11; CaCl2, 1, HEPES, 10, pH 7.2. To this was added 4 mM of lucifer yellow CH (dipotassium). Stock solutions of GABA, acetylcholine, and glutamic acid were diluted 1∶1000 on the day of use, and were applied by superfusion from a Y-tube [27].

Cells were recorded by conventional whole cell recording using an Alembic VE-2 amplifier (Alembic Instruments, Montreal, C. A. N.) operating at 100% series resistance compensation. Recordings were under the control of Pclamp v.7.0 (Molecular Devices, Sunnyvale, U. S. A.). In the search for h-currents, cells were held at −60 mV and stepped to −100 mV for one second, then back to −60 mV for one second. In the remaining voltage clamp recordings, the holding potential was −70 mV. Current-voltage curves were obtained by stepping the voltage from the holding potential to voltages of −80 to +50 mV in 10 mV increments. P/N leak subtraction (8 substeps) was used for all voltage step recordings. Current signals were filtered at 5 kHz and sampled at 25 kHz. Voltage signals were filtered at 2 kHz and sampled at 5 kHz.

## Results

### Assessment of dopaminergic inducing effect of feeder cells

**Figure 1:**
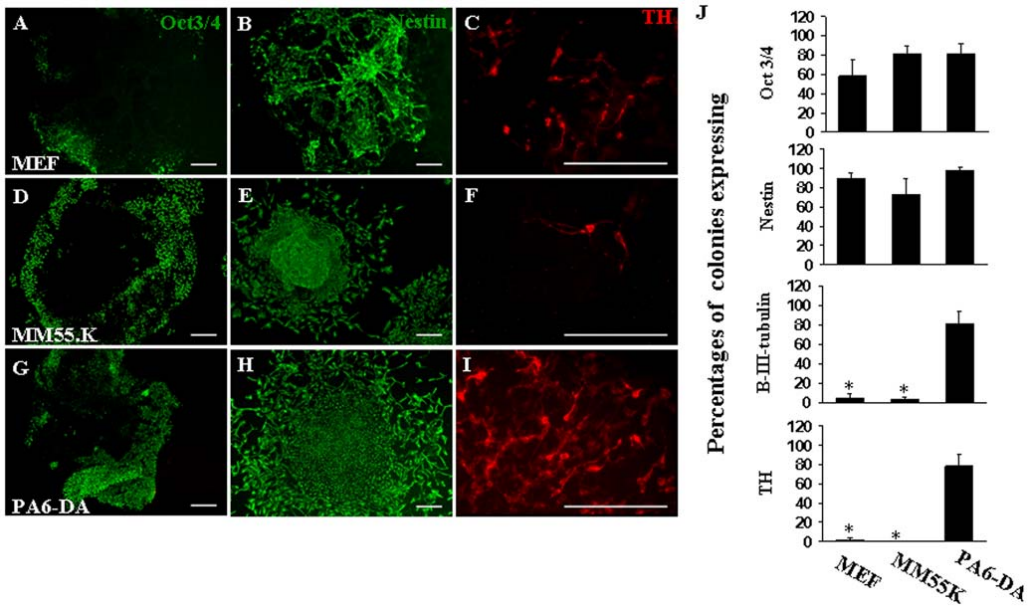
Marker expression analysis of differentiating hESC in co-cultures. BG01V2 colonies co-cultured with MEF (A–C), MM55K (D–F), and PA6-DA (G-I) for 12 days. Expression of the stem cell marker Oct3/4 was detected in more than half of the colonies grown of MEF (A), while 82% of colonies cultured on MM55K (D) and PA6-DA (G) contained Oct3/4+ cells. The early neuronal marker nestin was expressed in the majority of colonies in co-culture with MEF (B), MM55K (E), and PA6-DA (H) cells. The presence of dopaminergic neurons, identified by expression of TH, was found in 2% of colonies in MEF co-cultures (C) and in less than 1% of colonies grown on MM55K cells (F). The majority of colonies cultured on PA6-DA contained large numbers of TH+ neurons (I). Quantification of percentages of colonies expressing Oct3/4, Nestin, β-III-tubulin and TH after 12 days of co-culture on MEF, MM55K or PA6-DA feeder cells are presented as bar graphs with error bars indicating standard deviations. Data represent 60 colonies from three independent experiments (J). The overall difference in TH and β-III- tubulin expressing colonies was statistically significant in conditions with MEF and MM55K cells (P<0.0001), as compared to PA6-DA co-cultures. Scale bar = 200 µm.

In order to test the abilities of MEF, MM55K, MS5, PA6-X1, PA6-X and PA6-DA cell lines to induce DA induction of hESC, small colonies of BG01V2 were co-cultured with each of the feeder cell lines for 12 days. Undifferentiated stem cells were monitored by examining expression of Oct3/4 after 12 days (Fig. 1*A,D,G*). Percentages of Oct3/4+ colonies grown on MEF feeders and MM55K cells were 58±18% and 82±8% respectively (Fig. 1*A,D*) as compared to 82±10% colonies expressing Oct3/4+ in co-culture with the PA6-DA cells (Fig. 1*G*).

After 12 days, the majority of colonies grown on MEF, MM55K or PA6-DA cells contained cells expressing the immature neuroepithelial cell marker, nestin (Fig. 1*B,E,H*). Less than 1% of colonies cultured on MEF and MM55K contained cells immunoreactive for the DA neuronal marker TH (Fig. 1*C,F*). In contrast, 78±13% of colonies cultured on PA6-DA cells contained large numbers of TH+ neurons (Fig. 1*I*). Neurons expressing β-III-tubulin were detected in 5±5% and 3±3% of colonies grown on MEF and MM55K respectively, whereas co-culture with PA6-DA cells resulted in 82±13% β-III-tubulin+ colonies. The average percentages of Oct3/4, nestin, β-III-tubulin and TH expressing colonies cultured on MEF, MM55K and PA6-DA cells are presented as bar graphs in Figure 1*J*. The cell lines PA6-X1, PA6-X, and MS5 were unable to support the survival of hESC for 12 days. Therefore, we were not able to assess differentiation of hESC in co-culture with these three cell lines. MS5 cells, like PA6, are stromal cells and have been reported to promote midbrain-specific neural precursor differentiation [9], [10]. The MS5 cells used in this study were most likely a subtype of the original MS5 cell line, as they did not support DA differentiation. Additional information can be found at ftp://137.187.144.38/freed in the folder entitled “PA6 cell transformation”.

### Analysis of gene expression

By comparing gene expression of the PA6-DA cell line to the five cell lines lacking SDIA, we identified a set of candidate genes as potential DA-inducing elements. A complete gene list containing Z-ratios can be found in the supplemental data at ftp://137.187.144.38/freed. In total, 288 genes were preferentially expressed (Z-ratio≥3.0) in PA6-DA cells as compared to the PA6-X cell subtype. The majority of these genes were also significantly up-regulated in PA6-DA cells as compared to the transformed stromal cell lines, PA6-X1, MS5, and the MM55K and MEF cells. Biological processes were available for 159 of the 288 genes analyzed using the FatiGO web tool. The functions of these genes included 50 different categories at the most basic level.

**Figure 2:**
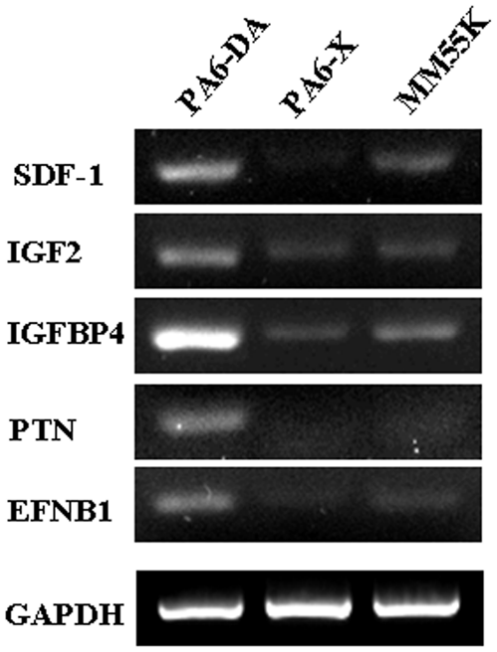
Confirmation of microarray results by RT-PCR. cDNA of PA6-DA, PA6-X and MM55K cell lines were analyzed by RT-PCR using gene specific primers for the five candidate genes selected from the microarray data. RT-PCR analysis reproduced the results of cDNA microarray for all five genes, including SDF-1, IGF2, IGFBP4, PTN, and EFNB1. Amplification was performed for 35 cycles. GAPDH was amplified simultaneously as an internal control under the same conditions.

In order to identify genes involved in biological processes and pathways related to CNS development among the 288 up-regulated genes, we performed a more advanced gene ontology analysis using the FatiGO web tool at specificity level six, as illustrated in Table 1. By focusing on soluble secretory molecules and gene products with a possible role in CNS development and regulation of neurogenesis, we identified stromal cell-derived factor 1 (SDF-1/CXCL12), pleiotrophin (PTN), insulin-like growth factor 2 (IGF2), insulin-like growth factor binding protein 4 (IGFBP4), and ephrin B1 (EFNB1) as factors potentially responsible for DA inducing activity. This combination of factors was termed “SPIE”, as it was eventually determined that IGFBP4 was unnecessary (see below). These genes were among the most up-regulated in PA6-DA cells as compared to PA6-X or MM55K cells, each having a seven-fold or greater Z-ratio. Relative expression of these genes in PA6-DA versus each cell type is presented in Table 2. Differences in these transcripts as determined by RT-PCR were strongly correlated with the microarray results (Fig. 2).

**Table 1:** Gene clusters highly expressed in PA6-DA cells.

Gene Ontology Biological Process at Level 6	Gene	UniGene Accession	Percentage of Genes	Z-ratios	
				PA6-DA	MM55K
Neurogenesis	IDB4	NM_031166.1	5.88	3.16	2.57
	EFNB1	NM_010110.2		8.56	7.43
	CXCL12	NM_013655.2		16.27	14.34
	TIMP2	NM_011594.2		3.32	1.66
	MYH10	NM_009382.2		9.62	5.18
	THY1	NM_175260.1		3.15	2.90
	RUNX1	NM_009821.1		4.28	4.71
	NOTCH1	NM_008714.2		3.02	3.99
Central nervous system development	OTX1	NM_011023.2	3.68	3.22	2.04
	IDB4	NM_031166.1		3.16	2.57
	CXCL12	NM_013655.2		16.27	14.34
	MYH10	NM_175260.1		3.15	2.90
	NOTCH1	NM_008714.2		3.02	3.99
Tissue development	MGLAP	NM_008597.2	3.68	9.42	1.40
	PTN	NM_008973.1		13.50	14.60
	SOX9	NM_011448		11.62	6.33
	OTOR	NM_020595		4.04	4.61
	NOTCH1	NM_008714.2		3.02	3.99
Wnt receptor signaling pathway	SFRP1	NM_013834.1	2.94	14.24	14.53
	SFRP2	NM_009144.1		3.16	3.09
	FRZB	NM_011356.2		3.44	2.56
	PPAP2B	NM_080555.1		3.37	7.36
Cellular morphogenesis during differentiation	EFNB1	NM_010110.2	2.94	8.56	7.43
	CXCL12	NM_013655.2		16.23	14.34
	THY1	NM_009382.2		9.62	5.18
	NOTCH1	NM_008714.2		3.02	3.99
Neural crest cell differentiation	EFNB1	NM_010110.2	2.21	8.56	7.43
	SEMA3F	NM_011349.2		4.66	0.40
	SOX9	NM_011448		11.62	6.33
Regulation of cell migration	CXCL12	NM_013655.2	2.21	16.23	14.34
	THY1	NM_009382.2		9.62	5.18
Cell fate specification	SOX9	NM_011448	2.21	11.62	6.33
	NOTCH1	NM_008714.2		3.02	3.99
Neural tube development	LTAP	NM_033509.2	0.74	3.21	2.18

Gene clusters categorized into biological processes at level six with relevance to various aspects of brain development and maintenance of central neurological processes. Expression levels of genes in PA6-DA as compared to PA6-X and MM55K cells are presented as Z-ratios.

**Table 2:** Relative expression of the five candidate genes.

Genes	UniGene Accession	Z-ratios				
		PA6-DA/PA6-X	PA6-DA/PA6-X1	PA6-DA/MS5	PA6-DA/MM55K	PA6-DA/MEF
CXCL12	NM 013655.2	16.27	16.92	15.08	14.34	9.96
EFNB1	NM 010110.2	8.56	9.26	8.40	7.43	7.01
PTN	NM 008973.1	13.50	14.58	12.17	14.60	5.70
IGF-II	NM 010514.1	13.19	13.83	11.63	13.52	3.28
IGFBP4	NM 010517.2	14.18	13.39	12.70	12.50	5.73

Gene expression in the PA6-DA cell line versus the cell lines PA6-X, PA6-X1, MS5, MM55K, and MEF, shown as Z-ratios.

As shown in Table 1, the selected genes SDF-1(CXCL12) and EFNB1 were included in the CNS and neuronal development category, while PTN was placed in the tissue development gene cluster by FatiGO. IGF2 and IGFBP4 were not included in categories related to neural development, but were assigned to organ morphogenesis and cell growth. In addition, PTN, Fibroblast growth factor-10 (FGF10), and serpin peptidase inhibitor, clade E (SERPINE2) also known as glial-derived neurite promoting factor, were classified as heparin binding factors by gene ontology in molecular function at level six. SERPINE2 and FGF10 were highly expressed in PA6-DA cells, with Z-ratios of approximately three and 14 respectively, as compared to both PA6-X and MM55K cells (Table S2).

### *In vitro* functional analysis of candidate molecules

To examine whether the selected molecules were directly responsible for SDIA, we initially used a previously established protocol [23] which was used to test the effects of PA6 cell conditioned medium. Undifferentiated hESC were simply transferred to hESC differentiation medium containing the five factors. Under these conditions, the majority of isolated hESC did not survive well or form colonies. To enhance cell survival, we generated EBs from hESC which were maintained in feeder-free conditions, after which the differentiation medium was replaced with hESC growth medium lacking the mitogen bFGF. After two-four days, resultant EBs were transferred to poly-L ornithine/laminin coated dishes and were allowed to differentiate in the presence of heparin and selected molecules.

When hESC maintained in feeder-free conditions were cultured in hESC medium lacking bFGF, but with omission of the EB step, they survived well, but SPIE induced minimal DA differentiation (data not shown).

**Figure 3:**
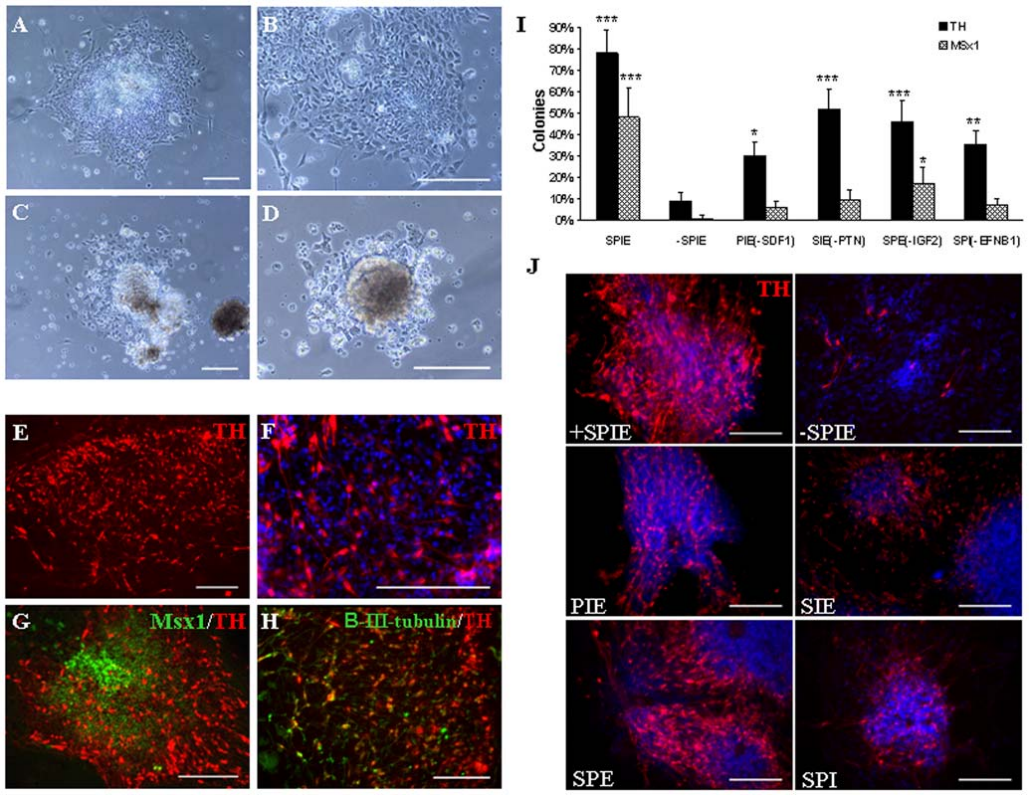
Differentiation of EBs into TH+ neurons induced by candidate molecules. Following EB formation, the hESC were transferred to poly-L-ornithine/laminin coated dishes and treated with SDF-1, IGF2, IGFBP4, PTN, EFNB1, and heparin. After three days, EBs attached to the plates and differentiated into neuroectodermal cells arranged in rosette-like structures with neuronal morphology (A, B). In contrast, the majority of EBs cultured in medium containing heparin in the absence of these molecules did not adhere. The few colonies that did attach in this condition exhibited exceedingly poor cell survival (C, D). After an additional seven days, the majority of colonies contained high percentages of TH+ neurons (E, F), although the center of colonies remained in a relatively undifferentiated state with expression of Msx1 (G). β-III-tubulin and TH co-labeling also revealed TH expression in the majority of neuronal cells (H). Scale bars = 200 µm. (I) Percentages of colonies expressing TH and Msx1 after 10 days under the influence of various combinations of the four factors, SDF-1(S), PTN (P), IGF2 (I), and EFNB1 (E). For numbers of TH-expressing colonies, each of the various combinations of three of the four factors was significantly different from both SPIE-treated and untreated cultures (all p<0.001 except untreated vs. SDF1 which was p<0.05 and SPIE vs. PTN which was p<0.01). Each of the combinations of three factors produced significantly fewer Msx1-positive colonies than did treatment with complete SPIE (all p<0.001). The percentage of Msx1-expressing colonies was significantly greater than the untreated condition only for the treatment with all four factors (SPIE) and for the condition excluding IGF2. * = P<0.05, ** = P<0.01, and *** = P<0.001 as compared to the untreated condition (Tukey-Kramer multiple comparisons following one-way analysis of variance). (J) Examples of colonies with omission of each individual factor, illustrating that fewer TH+ cells were detected within colonies, although numbers of TH+ cells within colonies could not be quantified.

The majority of EBs exposed to the selected factors in hESC culture medium formed radial glia scaffolds (Fig. 3*A*), and developed into cells in columnar arrangements after three days in culture (Fig. 3*B*). In contrast, less than 50% of EBs cultured in the hESC medium alone, in the presence of heparin, attached to the culture dishes. In addition, under the latter condition there was a substantial decrease in cell viability (Fig. 3*C,D*). To determine whether the cultures induced by these factors contained mesencephalic-restricted neural progenitor cells or DA neurons, analysis of Msx1 and TH expression was carried out after 10 days. The majority of colonies contained numerous TH+ neurons (Fig. 3*E, F*), predominantly found in the periphery of colonies. Cells in the central, denser areas of colonies were relatively undifferentiated at this time, as illustrated by expression of the midbrain progenitor marker Msx1 (Fig. 3*G*). TH was detected in the majority of β-III-tubulin-expressing cells (Fig. 3*H*). To further characterize the role of the five candidate molecules in TH+ cell induction, EB cultures were exposed to various combinations of inducing factors followed by immunocytochemical marker analysis after 10 days of differentiation. The results indicated that IGF2 enhanced survival of the proliferating NPC. In contrast, IGFBP4 reduced survival of the differentiating hESC, possibly by inhibiting IGF2 (data not shown).

The combination of SDF-1, PTN, IGF2, and EFNB1 was more efficient than the combination of five factors including IGFBP4. The combination of the four factors SDF-1, PTN, IGF2, and EFNB1 was therefore termed “SPIE”. The absence of any one component of SPIE resulted in a decrease in DA induction and differentiation, as compared to cultures exposed to complete SPIE (Fig. 3*I*). Omission of SDF-1 or EFNB1 decreased the number of TH+ colonies by more than 50%. Cultures lacking PTN or IGF2 contained approximately 33% and 41% less TH expressing colonies respectively. Exclusion of SDF-1 or EFNB1 also had the largest effect on generation of midbrain NPC as indicated by 86% and 85% decrease in Msx1+ colonies, respectively, as compared to treatment with all four factors (Fig. 3*I*). Exclusion of PTN or IGF2 decreased the percentage of colonies containing Msx1-expressing cells from 48%, to 17% and 10%, respectively. In addition, numbers of TH+ cells within colonies were greatly decreased in conditions lacking any one of the factors (Fig. 3*J*).

**Figure 4:**
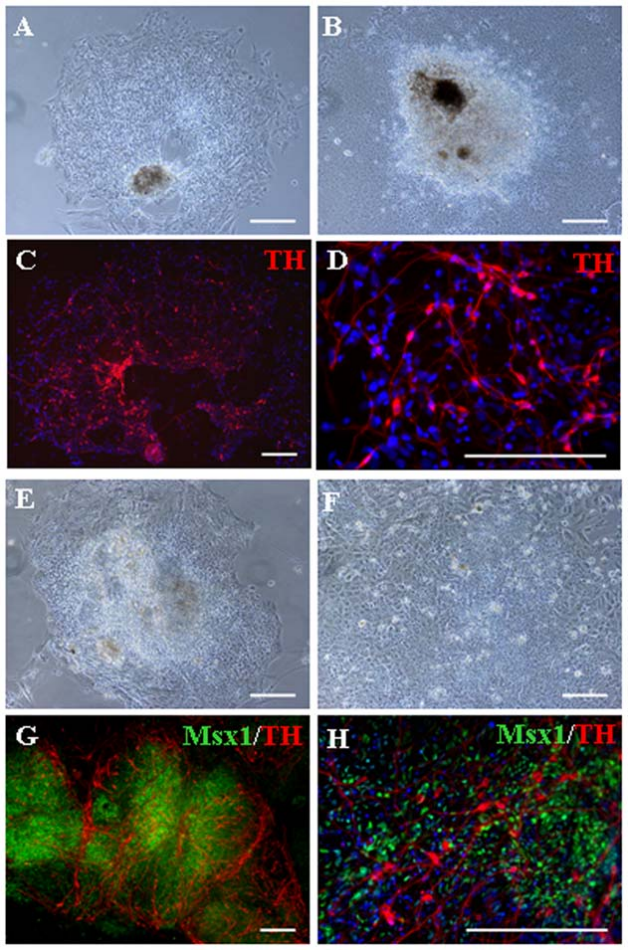
Effect of candidate molecules on differentiation of the BG02 and BG03 lines. Differentiation of EBs derived from the BG02 (A–D) and BG03 (E–H) hESC lines. Phase contrast images of (A) BG02-derived EBs differentiated in the presence of the candidate signaling molecules, SDF-1, IGF2, IGFBP4, PTN, EFNB1, or (B) in the absence of any factors, three days after seeding on adherent cultures. After 10 days of culture, TH-immunoreactive neurons were found in cultures treated with the five candidate molecules (C, D), and completely absent in the untreated cultures. Images of EBs generated from the BG03 cell line differentiating in the presence of candidate factors (E), or in cultures that did not contain any of the five molecules (F). (G, H) Expression of Msx1 and TH in BG03-derived cultures after 10 days of treatment with the selected factors, illustrating expression of Msx1 in the majority of cells within colonies. Scale bars = 200 µm.

To determine whether the synergistic action of the candidate molecules represents a general mechanism for the induction of DA neurons from hESC, we extended our analyses to two other hESC lines, BG02 and BG03, with normal karyotypes. EBs derived from the BG02 line were tested with all five factors, and showed overall poor survival. Colonies of differentiating BG02 cells in the presence of selected factors showed somewhat enhanced survival, and cells with a neuronal morphology were found within these colonies three days after EBs were transferred to adherent cultures (Fig. 4*A*). Conversely, cells within colonies that were grown in the absence of the molecules primarily had an epithelial morphology and did not contain cells with recognizable neuronal appearance (Fig. 4*B*). After five days of differentiation, TH+ neurons were found in treated cultures (Fig. 4*C, D*), but were completely absent from the untreated condition. Of note, expression of Msx1 was not found in either in the treated or the control condition for BG02 cells.

In contrast to BG02 cells, EBs derived from the BG03 cell line survived well either in the presence or absence of SPIE. The BG03-derived EBs that were exposed to SPIE differentiated into rosettes (Fig. 4*E*), morphologically similar to those seen in SDIA-induced BG01V2 cultures. In contrast, colonies in the untreated cultures had a more uniform appearance and fewer rosettes were seen in these cultures (Fig. 4*F*). When these EBs were allowed to differentiate for 10 days, more that 90% of colonies were Msx1+ with extensive networks of TH+ neuronal cells (Fig. 4*G*). Also, the majority of cells within colonies were Msx1+ (Fig. 4*H*)

### RT-PCR and immunoblot analysis of SPIE-treated cultures

**Figure 5:**
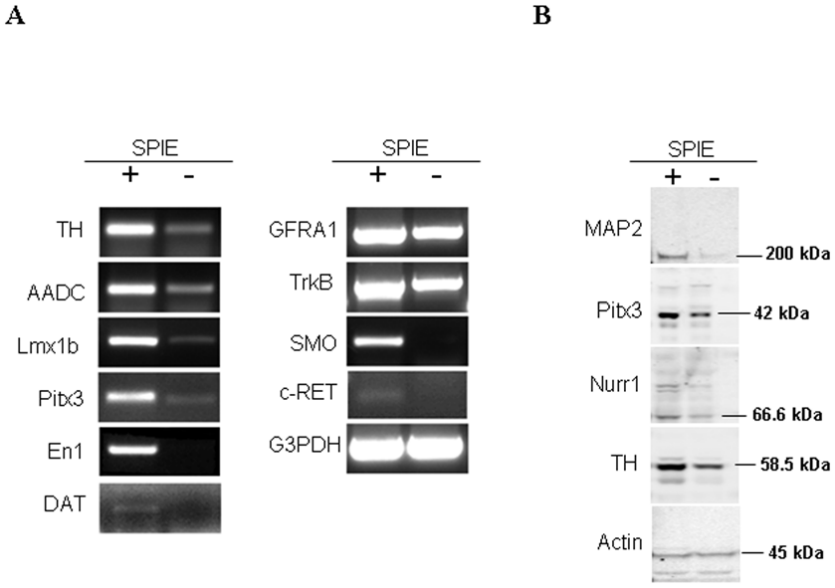
RT-PCR and western blot analysis of SPIE treated cultures. (A) Comparative RT-PCR results showing the effect of SPIE treatment on differentiation of BG01V2 cells to midbrain-specific DA neurons after 17 days, as confirmed by increased expression of TH, AADC, Lmx1b, Pitx3, En1, DAT and receptors including GFR1, TrkB, SMO, and c-RET. GAPDH was amplified under the same conditions (35 cycles) as an internal control. (B) Western blot analysis illustrating the increased levels of MAP2, Pitx3, Nurr1 and TH proteins, as compared to untreated cultures. The housekeeping gene actin shown in the lower panel was used as an internal loading control.

Expression of markers involved in midbrain DA neuron development were assessed by RT-PCR in SPIE-treated and untreated cultures after 17 days of culture (Fig. 5*A*). LIM homeobox transcription factor 1b (Lmx1b), and the enzymes of the dopamine synthetic pathway, TH and aromatic L-amino acid decarboxylase (AADC), were detected at substantially higher levels in cultures influenced by SPIE, as compared to randomly-differentiated cultures. Midbrain specific paired-like homeodomain transcription factor 3 (Pitx3) and engrailed 1 (En1) were expressed in SPIE-treated cultures, but not detected in untreated cultures. Expression of receptors GFR1 and c-RET for glial cell line-derived neurotrophic factor, was upregulated by SPIE. Increased expression of the brain-derived neurotrophic factor receptor, TrkB, and the smoothened (SMO) receptor was also confirmed in SPIE treated cultures. Neurons generated in the presence of SPIE also expressed the dopamine transporter (DAT) which is exclusively found in midbrain DA neurons.

Western blot analysis confirmed expression of the MAP2, Nuclear receptor 1 (Nurr1), and TH proteins (Fig. 5*B*). In agreement with previously obtained immunostaining data, TH and MAP2 levels were increased by SPIE treatment. Nurr1 was also detected in untreated cultures, but was increased by SPIE. The Pitx3 antibody detected a band at the expected molecular weight (42 kDa), which was increased in the SPIE-treated cultures (Fig. 5*B*). In addition, the Pitx3 antibody detected non-specific or unknown bands at approximately 95 and 145 kDa, and these bands were not increased by SPIE (data not shown).

### Electrophysiological studies

**Figure 6:**
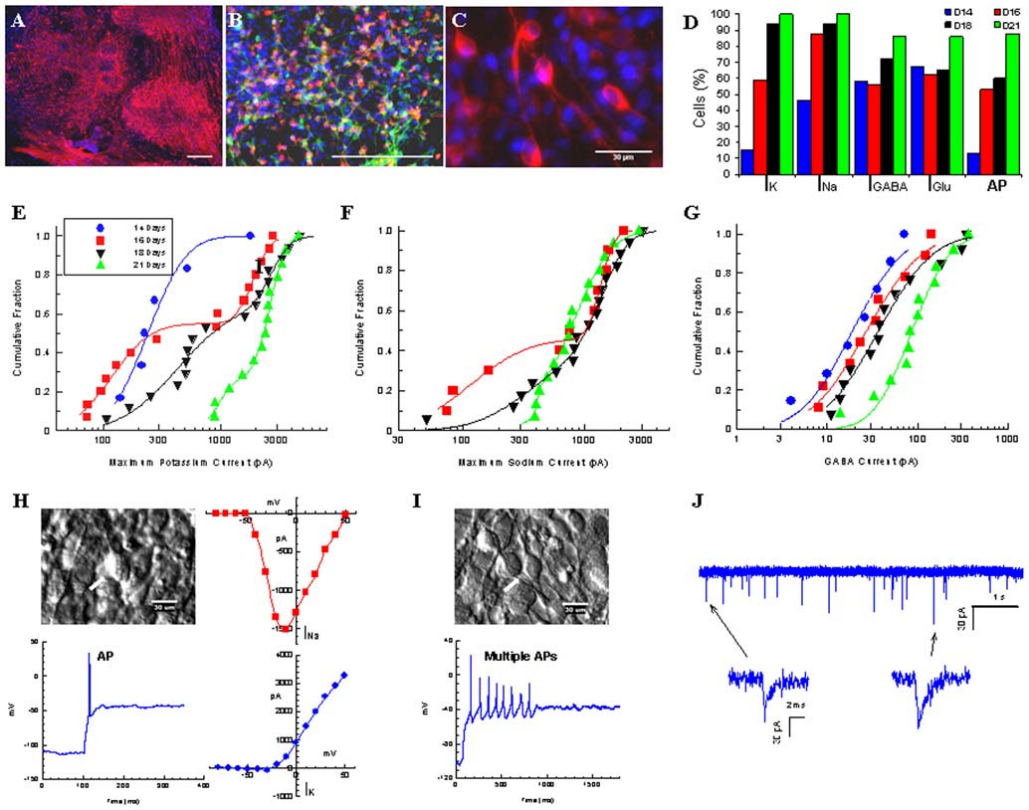
Electrophysiological analysis BG01V2-derived cultures differentiated by SPIE. Representative images of TH (A), and TH and MAP2 (B) immunocytochemistry of cultures differentiated for 21 days by SPIE (Scale bar 200 µm). Illustration of the cellular morphology of TH-immunoreactive neurons (C). Bar graph showing percentages of cells eliciting sodium (I_NA_), potassium (I_K_), GABA-evoked (I_GABA_), and Glutamate-evoked (I_Glu_) currents and percentage of cells capable of generating action potentials (AP) after 14 (blue), 16 (red), 18 (black) and 21 (green) days. Representation of cumulative log-normal distribution of maximum potassium currents (E), sodium currents (F), and GABA currents (G) over time. Hoffman modulation contrast image of a cells showing electrical excitability as indicated by strong inward sodium currents (red curve) and outward potassium currents (blue curve) under voltage clamp at day 21 of differentiation (H). In current clamp mode, this cell exhibited a strong AP which was obtained by a 90 pA current step from a membrane potential of −112 mV with an after hyperpolarization (H). An example of a neuron capable of generating multiple APs after 18 days of differentiation (I). Spontaneous, miniature postsynaptic currents recorded from a cell at day 21 of differentiation (J).

After 21 days of differentiation, about 80% of colonies contained numerous TH+ neurons (Fig. 6*A*). Cell counting revealed that 45±12% of the total number of cells were neurons, as determined by expression of the mature neuronal marker MAP2. Double staining showed co-localization of TH in 71±8% of MAP2+ neurons (Fig. 6*B*). We carefully assessed the cell morphology of TH+ cells which showed a fusiform shape typical of DA neurons [28] (Fig. 6*C*), and selected neurons with this morphological phenotype for electrophysiological recordings after 14, 16, 18, and 21 days of differentiation. The fraction of excitable cells, as measured by voltage gated sodium and potassium currents, and the fraction of cells showing sensitivity to the neurotransmitters GABA, and glutamate showed progressive changes (Fig. 6*D*).

The maximum current values for potassium (Fig. 6*E*) and sodium (Fig. 6*F*) generally had a bimodal (log-normal) distribution. For both currents, the fraction of the smaller mode shrank with time while the amplitude of the larger mode remained fairly constant. The GABA-induced currents also showed progressive increases with time (Fig. 6*G*). By contrast, 64 to 81% of the cells showed no response to acetylcholine (ACh), and this percentage showed no clear change with time. Even in responsive cells, the ACh-evoked currents were generally small (<20 pA). Currents elicited by glutamate showed a tendency to increase with time in culture, but evidence of a time-dependent trend was weak. In concert with the relatively large voltage gated currents was the appearance of single (Fig. 6*H*) and occasionally multiple action potentials (Fig. 6*I*) in current clamp mode. The increase in the percentage of cells that generated action potentials over time is illustrated in Figure 6*D*. None of the cells showed a hyperpolarization-activated h-current characteristic of dopamine neurons.

After 21 days of differentiation, spontaneous synaptic currents were detected (Fig. 6*J*).

Administration of tetrodotoxin did not reduce the amplitude or time course of these currents, indicating that they were spontaneous, miniature postsynaptic currents. The small amplitudes (22.6±8.4 pA) and brief decays (0.93±0.15 ms) imply that these currents were mediated by glutamate.

## Discussion

Over the past several years, there have been an increasing number of studies describing generation of DA neurons from mouse, primate and hESC [1]–[17]. These approaches, in most cases, involve co-culture systems using various feeder cells mediating neural and DA inductive signaling. Currently, the cell population with the greatest ability to support DA neuron differentiation is the mouse bone marrow-derived PA6 stromal cell line [8]–[13], [18], [23]. Alternatively, protocols for the enrichment of DA neurons from human and mouse ES cells without feeder cells require generation of retinoic acid treated EBs followed by exposure to midbrain specification factors such as SHH and FGF8 [2], [5], [18], [19].

Recently, transplantation of SDIA-derived neurons from monkey ESC was reported to result in an improvement in parkinsonian symptoms in a primate animal model [13]. Although such findings are encouraging, for clinical translation all cell-based therapeutic agents would need to be free of animal-derived cells or materials because of concerns about possible transfer of pathogens or xenogens, which may trigger immune reactions in human subjects.

When SDIA was identified by Kawasaki and coworkers [8], it was proposed that this activity accumulated on the cell surface. Although there was an indication that PA6 cells produced soluble inducing factors, significant induction of differentiation could not be achieved by PA6-conditioned medium alone [8]. In searching for possible mechanisms, this group and others have looked at BMP and Wnt signaling effects on neural differentiation [8], [29], but it has so far not been possible to reproduce the SDIA effect in the absence of PA6 cells. Previously we reported that the SDIA effect of PA6 cells resided in secreted factors, because conditioned medium had a clear DA-inducing effect in the presence of heparin.[23].

To better understand the molecular basis of SDIA, in the current study we compared gene expression array data sets developed by profiling different cell lines, including the potent PA6 line, to identify genes potentially responsible for early DA induction of hESC. We selected a combination of five transcripts that were highly expressed in the PA6-DA cells, including SDF-1, PTN IGF2, IGFBP4, and EFNB1, as potential DA-promoting elements. In the presence of these molecules, the majority of hESC-derived EBs differentiated into Msx1+ mesencephalic neural progenitor cells and TH+ neurons after 10–14 days, clearly mimicking the SDIA effect.

Application of the selected molecules to EBs derived from two karyotypically normal hESC lines, BG02 and BG03, provided further evidence that these compounds were indeed capable of initiating the process of DA differentiation. Although the three hESC lines tested with the selected factors, BG01V2, BG02, and BG03, showed substantial differences in their baseline degree of differentiation in the absence of the molecules, TH expression was consistently increased by SPIE treatment in all three lines. Importantly, the cellular morphology of the TH+ cells generated by SPIE (Fig. 6C) was similar to that of TH+ cells derived by the SDIA method [24].

The survival of the BG02 hESC line under these conditions was very limited as compared to that of the BG01V2 and BG03 cell lines, and treatment of BG02-derived EBs with SPIE did not result in expression of Msx1. We believe that there are differences in the nature of various hESC lines, and that BG02 might require different culture conditions, concentrations of factors, or additional extrinsic signaling to differentiate to midbrain specific DA neurons. Indeed, other studies have also reported a striking difference between neural and DA induction of various hESC on mouse stromal cell lines including PA6 and MS5 [3], [6]. We have also observed (unpublished data) that differences between BG01V2, BG02, and BG03 cells in DA differentiation capacity are similar to those seen in the present study when differentiated using SDIA or the method of Yan and coworkers [2].

We also assessed the individual contributions of each of the five factors and found that inclusion of IGFBP4 decreased the survival of differentiating midbrain NPC, and that IGFBP4 was not necessary for DA induction of hESC. IGF2 and PTN increased the number of surviving colonies and TH+ neurons, respectively, while SDF-1 and EFNB1 appeared to be required for specification of DA neurons. Thus, the combination of the four factors SDF-1, PTN, IGF2, and EFNB-1 termed “SPIE”, can to some degree mimic SDIA and produce a high yield of TH^+^ neurons from hESC in the presence of heparin.

Previously published Massively Parallel Signature Sequencing (MPSS) analyses of hESC gene expression [30]–[32] were examined to determine whether the SPIE receptors are expressed in hESC. The IGF2 receptor (IGF2R) was expressed in undifferentiated hESC. IGF2R expression was, however, negligible in hESC differentiated as EBs for three weeks [31], or differentiated on PA6 cells. Expression of ephrin B1 receptors EPHB2 and EPHB4 was also detected in undifferentiated hESC. Differentiation increased expression of EPHB2 and EPHB3. The PTN receptor N-syndecan (SDC3) was expressed in undifferentiated hESC as well as in hESC differentiated on PA6 cells [30]. The SDF-1 receptor, CXCR4, was undetectable or minimally expressed in undifferentiated hESC, although it should be noted that the form of MPSS used at that time failed to detect certain transcripts such as human TH [30]. In a previous study by our group, gene expression profiling by a focused human stem cell microarray showed expression of CXCR4 in neurons generated from hESC by the SDIA method [4]. In addition, previous focused gene expression array revealed that IGFR1, IGFR2 and CXCR4 were expressed in undifferentiated BG03 hESC [32]. A summary of the MPSS data can be found at ftp://137.187.144.38/freed in the file entitled “MPSS detection of SPIE receptors”. The varying levels of expression of these receptors suggests the possibility that the various components of SPIE may be required at different stages of differentiation, a possibility that we have not yet tested.

Induction of a midbrain DA phenotype by SPIE was confirmed by the expression of midbrain specific markers including Lmx1b, which is present during early specification of DA precursors [33], Pitx3 [34] and En1 [35]. In addition, expression of receptors GFR1 [36] and c-RET [37] for glial cell line-derived neurotrophic factor, which is a selective neurotrophic factor for midbrain DA neurons [38], was upregulated by SPIE. Increased expression of the brain-derived neurotrophic factor receptor, TrkB [39], [40], and the SMO receptor that is involved in SHH signaling [41], both of which are highly expressed in midbrain DA neurons, was also detected in SPIE-treated cultures. Furthermore, neurons differentiated from hESC by SPIE exhibited electrical excitability, generated action potentials, and spontaneous, miniature postsynaptic currents which reflects establishment of functional synaptic networks.

It is interesting that there are two apparently unrelated procedures for differentiating mesencephalic DA neurons from hESC; techniques involving SDIA or SPIE, and the method of Yan and coworkers [2]. Although both SPIE and the Yan et. al. [2] techniques employ an EB formation phase, the treatment protocols and growth factors which are employed are quite different. In the present study, the morphology of SPIE-derived cells was similar to that of cells differentiated by SDIA [24] and to that of DA neurons differentiated by the procedure described by Yan and coworkers [2]. Cells produced by the two methods are also similar in that they express markers of mesencephalic DA neurons. There may, however, be other or more subtle differences between the cells derived by the two methods, which would require additional studies to identify.

### Growth and soluble factors produced by PA6 cells

Heparan-sulfate glycosaminoglycans such as heparin exist at the cell surface and also in the extracellular matrix of many different cell types are known to interact strongly with several growth factors and modulate their biological activity during development [42]–[44]. PTN is among the factors reported to have a high affinity association with heparin-like molecules [45]. PTN has trophic effects on DA neurons and increases the yield of TH+ neurons differentiated from mouse ESC [46].

Serpin peptidase inhibitor, clade E, also known as SERPINE2, was also identified among the heparin binding factors that were differentially expressed in PA6-DA cells. SERPINE2 protein is highly expressed in a variety of brain regions including the striatum during nervous system development [47]. SERPINE2 was up-regulated, by a Z-ratio of more than three, in PA6-DA cells in comparison with the transformed PA6, MS5, and MM55K cells. However, PA6-DA and MEF cells showed nearly equal expression of SERPINE2. This gene product was not tested for neural or DA induction of hESC.

Another heparin binding factor, differentially expressed by PA6 cells was FGF-10, which is not considered to have a role in CNS development. Nonetheless the introduction of FGF10 to chick embryos up-regulated FGF8 expression in the ectoderm through Wnt3a signaling and stimulated SHH expression in the posterior mesoderm followed by outgrowth initiation of chick limb structures [48], [49]. These interactions of FGF10 with well-known midbrain patterning cues suggests that this factor may be involved in FGF8 and SHH signaling to enhance DA neurogenesis. Effects of FGF10 on DA induction were also not tested.

The chemokine SDF-1 was originally identified in the immune system and induces leukocyte chemotaxis [50]. In addition to its T-cell chemotactic activity, SDF-1 is widely expressed in the nervous system, and has been proposed to have a role in cerebellar development [51], [52]. SDF-1 also interacts with the Wnt pathway in neural development [53] and mediates sonic hedgehog-induced proliferation of cerebellar granule cells [54]. Up-regulation of SDF-1 and its receptor CXCR4 was also observed during differentiation of neural stem cells into more restricted precursors [55]. Interestingly, a very recent study by Edman and colleagues [54] demonstrated expression of two other α-chemokines, CXCL1 and CXCL6, in developing rodent ventral midbrain and suggested that these factors have a regulatory role in the development of the midbrain DA cell population.

IGFs and their carriers, IGF-binding proteins (IGFBPs) are widely expressed throughout the CNS [56]–[58]. IGF2 has been suggested to have neurotrophic effects, promoting survival and differentiation of neuronal cells [59], [60]. An indication of the involvement of IGF2 in differentiation of mesencephalic neural progenitor cells from hESC was recently obtained by our previous MPSS analysis of gene expression in PSA-NCAM+ neuronal precursors derived by SDIA. The IGF2 and H19 transcripts were most abundant of the 11,912 distinct sequences detected. IGF2 expression was also found in laser-captured dopamine neurons from human postmortem brain [30].

Transport and bioactivity of IGFs are regulated by six IGF-binding proteins (IGFBP1-6) [61]. In addition to IGF2, we found elevated expression of IGFBP4 in PA6-DA cells. Although there are reports of elevation of IGFBP4 expression in rodents [62] and neural precursors enriched from the human fetus during brain development [63], the physiological role and signaling pathways of IGFBP4 in neuronal differentiation are unknown. Co-localization of IGF2 and IGFBP4 mRNAs as revealed by in situ hybridization studies suggests a correlation between these two proteins in the developing embryo [64]. We believe that involvement of IGFBP4 in the SDIA effect may be linked to regulation of IGF2 activity, and in our study, IGFBP4 seemed to decrease the survival of differentiating NPC.

Eph receptors and their ephrin ligands are essential for migration and cell interactions of many cell types during embryonic development [65], [66]. B-class ephrins are transmembrane proteins which bind EphB receptors. Ephrin B1 acts both as a ligand and as a receptor in a tissue-specific manner during embryogenesis and is critically important in many biological processes including axon guidance, neural crest migration [66], [67]. The role of ephrin B1 in directing neuronal patterning in neural stem cells has not been tested. However, another member of the ephrin family, ephrin B2, and its receptor EphB1, have been found in midbrain DA neuronal cells, where ephrin B2 induced cell loss of substantia nigra, but not ventral tegmental DA neurons, suggesting a role of this ligand-receptor pair in specification of the nigrostriatal pathway [68]. In our previous study where the contributions of cell surface and soluble factors to the SDIA effect was examined, we found that PA6 cell surface factors promoted cell survival and enhanced overall neurogenesis of hESC, rather than providing lineage-specific instructions. Nevertheless, the presence of cell surface factors enhanced the survival of hESC during differentiation, which led to an increase in the overall yield of DA neurons [23]. Also, DA differentiation in cultures of hESC treated with factors secreted from PA6 cells plus heparin was relatively less effective as compared to co-cultures with intact PA6 cells [23]. This discrepancy may be due to a progressive loss of activity of the secreted signaling factors. Also, when PA6 cell surfaces were tested for SDIA, paraformaldehyde was used to kill the PA6 cells [23]. The fixation may have damaged membrane bound factors or the extracellular domain of transmembrane proteins such as ephrin B1, masking the potential role of these molecules in DA differentiation. Thus, although ephrin B1 is not a soluble secreted factor, we chose to examine its role in DA neurogenesis.

The combination of four factors, currently termed SPIE, was able to reproduce the effect of SDIA, however, the possibility that additional signaling molecules would further enhance DA induction from hESC cannot be excluded. Several other factors were up-regulated, but to a significantly lesser degree. These included members of the TGF-β family and factors involved in the Wnt signaling pathway. It has been suggested that these factors play a critical role in controlling neuronal fate and the establishment of the DA phenotype [69], [70]. It also may be that additional factors would need to be added to SPIE to avoid the requirement for the use of an EB induction phase, as was employed in the current protocol.

It should also be noted that we detected significantly higher levels of the soluble Wnt inhibitor secreted Frizzled-related protein 1(sFRP-1) in PA6-DA cells, which is suggested to have an inhibitory effect on ventral midbrain neuronal development (Z-ratio>14) than sFRP-2, which promotes DA neurogenesis and maturation (Z-ratio>3) [71]. On the other hand, these soluble factors might modulate the Wnt signaling pathway in a different way during earlier stages of development, and thus could also play a role in SDIA.

In summary, our findings constitute a simple system for the induction and differentiation of hESC into DA neurons in a culture system free of xenogenic cells or material. Future investigations of the biological effect and mechanism of action of these factors is likely to increase our understanding of the molecular signaling that controls DA neuron differentiation from hESC, ultimately contributing to the advancement of cellular replacement strategies for treatment of PD. It should also be mentioned that the risk of tumor formation arising from an undifferentiated population of transplanted cells would need to be assessed, and methods enhancing post-implantation cell survival would need to be developed, before SPIE could be employed as a transplantation strategy.

## Supporting Information

Table S1 PCR primer sets.
(0.06 MB DOC)

Table S2 Genes differentially expressed in PA6-DA cells. The 288 genes highly expressed in PA6-DA cells as compared to the PA6-X cell subtype (Z-ratio≥3.0). The table shows relative expression of these genes in PA6-DA cells as compared to the transformed stromal cell lines, PA6-X1 and MS5, and to the MM55K and MEF cells, as Z-ratios.
(0.56 MB DOC)
