# Supplementary material for: A Novel Combination of Factors, Termed SPIE, which Promotes Dopaminergic Neuron Differentiation from Human Embryonic Stem Cells
Source: PLoS One. 2009 Aug 12;4(8):e6606. doi: 10.1371/journal.pone.0006606 (PMC2719871; doi:10.1371/journal.pone.0006606)
Supplement: Table S1 — PCR primer sets. (0.06 MB DOC) [file pone.0006606.s001.doc]

**Table S1.** PCR primer sets

***Mouse Primer Sets***

| **Gene** | **Primer Bank ID** | **Forward** | **Reverse** |
| --- | --- | --- | --- |
| IGF2 | 6754310a1 | GTGCTGCATCGCTGCTTAC | ACGTCCCTCTCGGACTTGG |
| PTN | 6679543a1 | ATGTCGTCCCAGCAATATCAGC | CCAAGATGAAAATCAATGCCAGG |
| CXCL12 | 7305465a1 | TGCATCAGTGACGGTAAACCA | TTCTTCAGCCGTGCAACAATC |
| EFNB1 | 6753726a1 | TGTGGCTATGGTCGTGCTG | CCAAGCCCTTCCCACTTAGG |
| IGFBP4 | 6981086a1 | AGAAGCCCCTGCGTACATTG | TGTCCCCACGATCTTCATCTT |
| RBP1 | 6755300a1 | CTGAGCAATGAGAATTTCGAGGA | GCGGTCGTCTATGCCTGTC |
| VCAM1 | 31981430a1 | AGTTGGGGATTCGGTTGTTCT | CCCCTCATTCCTTACCACCC |
| Adamts5 | 6752976a1 | GGAGCGAGGCCATTTACAAC | CGTAGACAAGGTAGCCCACTTT |
| DCN | 6681143a1 | TCTTGGGCTGGACCATTTGAA | CATCGGTAGGGGCACATAGA |
| COL1A2 | 6680980a1 | GTAACTTCGTGCCTAGCAACA | CCTTTGTCAGAATACTGAGCAG |

***Human Primer Sets***

| **Gene** | **Forward** | **Reverse** |
| --- | --- | --- |
| Lmx1b | AACTGTACTGCAAACAAGACTACC | TTCATGTCCCCATCTTCATCCTC |
| AADC | GGGACCACAACA TGCTGCTC | CCACTCCATTCAGAAGGTGCC |
| TH | TCATCACCTGGTCACCAAGTT | GGTCGCCGTGCCTGTACT |
| TrkB | AGGGCAACCCGCCCACGGAA | TTGGTGGCCTCCAGCGGCAG |
| En1 | CTGGGTGTACTGCACACGTTAT | TACTCGCTCTCGTCTTTGTCCT |
| Pitx3 | AGCTGCCTTTGCATAGCTCG | AGCTGCCTTTGCATAGCTCG |
| Smo | TATTCACTCCCGCACCAAC | AGCCAGACATCCAGAACTC |
| DAT | TTTCTCCTGTCCGTCATTGGC | AGCCCACACCTTTCAGTATGG |
| c-RET | CGACCTCATCTCATTTGCC | AATCTTCATCTTCCGCCCC |
| GFRA1 | AGGGAAATGATCTGCTGGAGGA | CTCTGGCTG GCAGTTGGTAAAA |
| GAPDH | ACCACAGTCCATGCCATCAC | TCCACCACCCTGTTGCTGTA |
