# Supplementary material for: A Novel Combination of Factors, Termed SPIE, which Promotes Dopaminergic Neuron Differentiation from Human Embryonic Stem Cells
Source: PLoS One. 2009 Aug 12;4(8):e6606. doi: 10.1371/journal.pone.0006606 (PMC2719871; doi:10.1371/journal.pone.0006606)
Supplement: Table S2 — Genes differentially expressed in PA6-DA cells. The 288 genes highly expressed in PA6-DA cells as compared to the PA6-X cell subtype (Z-ratio≥3.0). The table shows relative expression of these genes in PA6-DA cells as compared to the transformed stromal cell lines, PA6-X1 and MS5, and to the MM55K and MEF cells, as Z-ratios. (0.56 MB DOC) [file pone.0006606.s002.doc]

**Table S2.** Genes differentially expressed in PA6-DA cells

| **Accession** | **Symbol** | **Definition** | **PA6-DA/ PA6-X** | **PA6-DA/ PA6-X1** | **PA6-DA/ MS5** | **PA6-DA/ MM55K** | **PA6-DA/ MEF** |
| --- | --- | --- | --- | --- | --- | --- | --- |
| NM_013655.2 | CXCL12 | Mus musculus chemokine (C-X-C motif) ligand 12 (Cxcl12), transcript variant 2, mRNA. | 16.26702509 | 16.92234365 | 15.07725664 | 14.33695921 | 9.957243441 |
| NM_013834.1 | SFRP1 | Mus musculus secreted frizzled-related sequence protein 1 (Sfrp1), mRNA. | 14.23530837 | 15.33646051 | 13.28748094 | 14.52614099 | 8.90656643 |
| NM_010517.2 | IGFBP4 | Mus musculus insulin-like growth factor binding protein 4 (Igfbp4), mRNA. | 14.17673242 | 13.39014083 | 12.70405318 | 12.49805636 | 5.731494826 |
| NM_019521.1 | GAS6 | Mus musculus growth arrest specific 6 (Gas6), mRNA. | 13.61539754 | 12.98114194 | 12.88564658 | 13.31336886 | 6.450231352 |
| NM_008973.1 | PTN | Mus musculus pleiotrophin (Ptn), mRNA. | 13.49581098 | 14.57821373 | 12.16670194 | 14.59559389 | 5.699670041 |
| NM_011595.1 | TIMP3 | Mus musculus tissue inhibitor of metalloproteinase 3 (Timp3), mRNA. | 13.43738782 | 10.57710653 | 12.36487219 | 3.077008714 | 7.718972348 |
| NM_010514.1 | IGF2 | Mus musculus insulin-like growth factor 2 (Igf2), mRNA. | 13.18578687 | 13.82533546 | 11.63295054 | 13.5245862 | 3.279469822 |
| NM_007743.1 | COL1A2 | Mus musculus procollagen, type I, alpha 2 (Col1a2), mRNA. | 13.08678896 | 11.64257357 | 11.95232385 | 5.305717573 | 5.653380713 |
| NM_011254.2 | RBP1 | Mus musculus retinol binding protein 1, cellular (Rbp1), mRNA. | 12.90027114 | 11.12214343 | 11.92705798 | 5.045499339 | 5.703124899 |
| NM_010052.1 | DLK1 | Mus musculus delta-like 1 homolog (Drosophila) (Dlk1), mRNA. | 12.52321689 | 13.90746764 | 11.89771956 | 13.71246854 | 11.19278454 |
| NM_010517.2 | IGFBP4 | Mus musculus insulin-like growth factor binding protein 4 (Igfbp4), mRNA. | 11.9828293 | 11.62914637 | 10.97121313 | 12.15124972 | 6.324636207 |
| NM_022316.1 | SMOC1 | Mus musculus SPARC related modular calcium binding 1 (Smoc1), mRNA. | 11.66979611 | 9.991773641 | 10.38686747 | 5.796863141 | 11.00286342 |
| NM_011448 | SOX9 | Mus musculus SRY-box containing gene 9 (Sox9), mRNA. | 11.61655762 | 11.67533074 | 10.9042318 | 6.333124937 | 7.876530507 |
| NM_021704.1 | CXCL12 | Mus musculus chemokine (C-X-C motif) ligand 12 (Cxcl12), transcript variant 2, mRNA. | 11.60438467 | 12.4408842 | 10.92371486 | 11.1182495 | 10.03852074 |
| NM_007833.1 | DCN | Mus musculus decorin (Dcn), mRNA. | 11.46844699 | 7.186446462 | 8.036511995 | 0.23431176 | 0.568211968 |
| NM_008695.1 | NID2 | Mus musculus nidogen 2 (Nid2), mRNA. | 11.32679885 | 10.478348 | 10.6064326 | 5.243150289 | 6.830142797 |
| NM_054077.2 | PRELP | Mus musculus proline arginine-rich end leucine-rich repeat (Prelp), mRNA. | 10.76365686 | 6.232935558 | 11.94527993 | 1.885314339 | 7.378806304 |
| NM_016854.1 | PPP1R3C | Mus musculus protein phosphatase 1, regulatory (inhibitor) subunit 3C (Ppp1r3c), mRNA. | 10.66959831 | 11.68302302 | 10.30759947 | 10.18458489 | 8.838222918 |
| NM_207229 | MGC41689 | Mus musculus hypothetical LOC211623 (MGC41689), mRNA. | 10.27142549 | 11.22534432 | 9.73400572 | 12.22028002 | 11.51414001 |
| NM_010330.2 | EMB | Mus musculus embigin (Emb), mRNA. | 9.84922808 | 10.45154636 | 8.919378509 | 8.605190114 | 3.509698189 |
| NM_009382.2 | THY1 | Mus musculus thymus cell antigen 1, theta (Thy1), mRNA. | 9.623577697 | 9.311885334 | 8.521260283 | 5.177757426 | 0.704510915 |
| NM_010211.1 | FHL1 | Mus musculus four and a half LIM domains 1 (Fhl1), mRNA. | 9.606526135 | 9.202079071 | 8.961491538 | 4.884049569 | 5.264141823 |
| NM_024283.1 | 1500015O10RIK | Mus musculus RIKEN cDNA 1500015O10 gene (1500015O10Rik), mRNA. | 9.542427556 | 8.304658061 | 8.963189917 | 2.565491965 | 5.289007447 |
| XM_283765.2 | 5430433G21RIK | Mus musculus RIKEN cDNA 5430433G21 gene (5430433G21Rik), mRNA. | 9.503812083 | 10.30250916 | 9.464551418 | 9.136379554 | 10.18236923 |
| NM_013592.2 | MATN4 | Mus musculus matrilin 4 (Matn4), mRNA. | 9.490792613 | 10.43578092 | 9.515619103 | 12.95067503 | 11.49371017 |
| NM_008597.2 | MGLAP | Mus musculus matrix gamma-carboxyglutamate (gla) protein (Mglap), mRNA. | 9.422036446 | 6.464602941 | 8.713408151 | 1.400481302 | 1.251053409 |
| NM_007833.1 | DCN | Mus musculus decorin (Dcn), mRNA. | 9.211747471 | 6.324829528 | 7.498189416 | 0.308177555 | 0.829597789 |
| NM_010549.1 | IL11RA1 | Mus musculus interleukin 11 receptor, alpha chain 1 (Il11ra1), mRNA. | 9.197041395 | 8.227197933 | 7.544085811 | 6.455348286 | 3.139011468 |
| NM_009349 | TEMT | Mus musculus thioether S-methyltransferase (Temt), mRNA. | 9.097453025 | 10.34319469 | 8.889645651 | 9.453124222 | 10.49755175 |
| NM_028903.1 | 4933425F03RIK | Mus musculus RIKEN cDNA 4933425F03 gene (4933425F03Rik), mRNA. | 8.663568498 | 9.202879317 | 8.243727149 | 7.115061285 | 9.453752196 |
| NM_054049 | OSR2 | Mus musculus odd-skipped related 2 (Drosophila) (Osr2), mRNA. | 8.642236682 | 8.872472875 | 8.152598448 | 5.791453001 | 6.346710859 |
| NM_010110.2 | EFNB1 | Mus musculus ephrin B1 (Efnb1), mRNA. | 8.561566572 | 9.256703812 | 8.396501219 | 7.432455178 | 7.005840761 |
| NM_009626.2 | ADH7 | Mus musculus alcohol dehydrogenase 7 (class IV), mu or sigma polypeptide (Adh7), mRNA. | 8.412368254 | 9.22575094 | 7.872952721 | 9.620070505 | 9.495348029 |
| NM_010550 | IL11RA2 | Mus musculus interleukin 11 receptor, alpha chain 2 (Il11ra2), mRNA. | 8.338525792 | 7.245731324 | 7.198747928 | 5.507368229 | 2.782080901 |
| NM_026439.1 | 2610001E17RIK | Mus musculus RIKEN cDNA 2610001E17 gene (2610001E17Rik), mRNA. | 8.157258928 | 6.598428069 | 8.591192969 | 1.895372079 | 2.825133346 |
| NM_031397.1 | BICC1 | Mus musculus bicaudal C homolog 1 (Drosophila) (Bicc1), mRNA. | 7.965391811 | 7.73295444 | 8.74102061 | 2.990706876 | 6.541013036 |
| NM_148938.2 | SLC1A3 | Mus musculus solute carrier family 1 (glial high affinity glutamate transporter), member 3 (Slc1a3), mRNA. | 7.838610993 | 8.97818777 | 7.518074688 | 9.136128311 | 7.150591325 |
| XM_127627.2 | PLAC9 |  | 7.678466139 | 8.70890155 | 7.481613798 | 9.511293583 | 8.95386099 |
| NM_172604.1 | SCARA3 | Mus musculus scavenger receptor class A, member 3 (Scara3), mRNA. | 7.49516884 | 7.273247404 | 6.963988587 | 2.887346465 | 3.583960507 |
| NM_008002.3 | FGF10 | Mus musculus fibroblast growth factor 10 (Fgf10), mRNA. | 7.42243108 | 7.787325142 | 6.861415839 | 6.850162673 | 6.647039401 |
| NM_007833.1 | DCN | Mus musculus decorin (Dcn), mRNA. | 7.377893 | 5.6111493 | 6.404630785 | 0.476684384 | 0.406775326 |
| NM_025817.3 | 1200009O22RIK | Mus musculus RIKEN cDNA 1200009O22 gene (1200009O22Rik), mRNA. | 7.290197398 | 7.577784417 | 6.63310679 | 7.375203048 | 6.974593859 |
| NM_013560.1 | HSPB1 | Mus musculus heat shock protein 1 (Hspb1), mRNA. | 7.119884726 | 5.388376381 | 5.763034651 | 0.536605349 | 1.397757357 |
| NM_019444.1 | RAMP2 | Mus musculus receptor (calcitonin) activity modifying protein 2 (Ramp2), mRNA. | 7.048582458 | 6.142066172 | 7.229526119 | 7.822602825 | 6.957677812 |
| NM_009025.1 | RASA3 | Mus musculus RAS p21 protein activator 3 (Rasa3), mRNA. | 6.993866933 | 1.963040997 | 2.470788571 | 3.002466032 | 2.181467943 |
|  | GCAP27 |  | 6.829207491 | 5.362354143 | 6.12042028 | 0.560202208 | 0.060782002 |
| NM_011576.1 | TFPI | Mus musculus tissue factor pathway inhibitor (Tfpi), mRNA. | 6.717615115 | 7.278570211 | 6.278348939 | 6.369085656 | 5.044897994 |
| NM_010216.1 | FIGF | Mus musculus c-fos induced growth factor (Figf), mRNA. | 6.541208765 | 6.7527078 | 6.951680864 | 6.720708409 | 6.926741118 |
| NM_146161.1 | ARHGAP24 | Mus musculus Rho GTPase activating protein 24 (Arhgap24), mRNA. | 6.471202437 | 6.535777356 | 5.93662682 | 4.364110805 | 3.039600625 |
| NM_030206.1 | CYGB | Mus musculus cytoglobin (Cygb), mRNA. | 6.395698181 | 5.193226354 | 6.843958469 | 4.848485157 | 9.55540283 |
| NM_009675.1 | AOC3 | Mus musculus amine oxidase, copper containing 3 (Aoc3), mRNA. | 6.372093318 | 5.310198337 | 6.947382243 | 11.96552804 | 8.743898073 |
| XM_123188.1 | COX7A2L | Mus musculus cytochrome c oxidase subunit VIIa polypeptide 2-like (Cox7a2l), mRNA. | 6.285998362 | 6.216346525 | 5.79760029 | 6.500612234 | 7.954053214 |
| NM_009964.1 | CRYAB | Mus musculus crystallin, alpha B (Cryab), mRNA. | 6.187205189 | 3.969234255 | 7.051643394 | 1.350501542 | 2.322867351 |
| NM_176922.4 | ITGA11 | Mus musculus integrin, alpha 11 (Itga11), mRNA. | 6.171380988 | 5.280124971 | 4.708229852 | 0.084278865 | 4.326636495 |
| NM_010656.1 | SSPN | Mus musculus sarcospan (Sspn), mRNA. | 6.142440165 | 6.884444405 | 5.7477803 | 7.11510146 | 6.974138094 |
| NM_172119 | DIO3 | Mus musculus deiodinase, iodothyronine type III (Dio3), mRNA. | 6.138408076 | 7.009906829 | 6.062639127 | 7.315601938 | 6.755201765 |
| NM_009778.1 | C3 | Mus musculus complement component 3 (C3), mRNA. | 6.098293219 | 2.955794212 | 10.91521232 | 2.745988093 | 14.33752824 |
| NM_030143.2 | DDIT4L | Mus musculus DNA-damage-inducible transcript 4-like (Ddit4l), mRNA. | 6.079583976 | 6.855868829 | 5.814328782 | 6.492859912 | 4.281704779 |
| NM_008760.2 | OGN | Mus musculus osteoglycin (Ogn), mRNA. | 6.045735718 | 6.224037119 | 6.878874885 | 4.781958058 | 7.967088962 |
| NM_007409.2 | ADH1 | Mus musculus alcohol dehydrogenase 1 (class I) (Adh1), mRNA. | 6.023657061 | 6.519364016 | 5.759349255 | 6.86040422 | 4.033333455 |
| NM_016794.2 | VAMP8 | Mus musculus vesicle-associated membrane protein 8 (Vamp8), mRNA. | 5.946526761 | 3.535844522 | 1.517344967 | 1.962743779 | 0.370594634 |
| NM_153393.1 | COL23A1 | Mus musculus procollagen, type XXIII, alpha 1 (Col23a1), mRNA. | 5.936526075 | 4.380879753 | 3.615321583 | 4.758891192 | 3.8874406 |
| NM_007791.2 | CSRP1 | Mus musculus cysteine and glycine-rich protein 1 (Csrp1), mRNA. | 5.936329253 | 4.620515759 | 5.563496403 | 0.625774708 | 4.298480686 |
| NM_011413 | SLP | Mus musculus sex-limited protein (Slp), mRNA. | 5.890530756 | 5.532284522 | 5.758015594 | 6.732281282 | 6.697906391 |
| NM_016658.1 | GALT | Mus musculus galactose-1-phosphate uridyl transferase (Galt), mRNA. | 5.768907499 | 5.57162054 | 4.474849278 | 4.064619119 | 2.770822793 |
| NM_025311.1 | D14ERTD449E | Mus musculus DNA segment, Chr 14, ERATO Doi 449, expressed (D14Ertd449e), mRNA. | 5.758852897 | 5.640575184 | 4.907370038 | 4.792066976 | 4.614444569 |
| NM_008760.2 | OGN | Mus musculus osteoglycin (Ogn), mRNA. | 5.752878892 | 4.776900379 | 6.094266295 | 3.829214203 | 6.671728161 |
| NM_016865.2 | HTATIP2 | Mus musculus HIV-1 tat interactive protein 2, homolog (human) (Htatip2), mRNA. | 5.748139032 | 5.286285846 | 5.424963518 | 1.454011093 | 1.861223412 |
| XM_194207.3 | 3732412D22RIK | Mus musculus RIKEN cDNA 3732412D22 gene (3732412D22Rik), mRNA. | 5.743303587 | 6.278039393 | 5.466340661 | 5.611993672 | 6.27920647 |
| NM_009964.1 | CRYAB | Mus musculus crystallin, alpha B (Cryab), mRNA. | 6.187205189 | 3.969234255 | 7.051643394 | 1.350501542 | 2.322867351 |
| XM_358343.1 | SULF2 |  | 5.704314819 | 5.021212637 | 4.965757456 | 1.464283733 | 1.907561819 |
| NM_011665.2 | UBE2I | Mus musculus ubiquitin-conjugating enzyme E2I (Ube2i), mRNA. | 5.697609261 | 6.299321577 | 5.11363843 | 6.298837484 | 4.501037305 |
| XM_355911.1 | PACE4 | Mus musculus paired basic amino acid cleaving system 4 (Pace4), mRNA. | 5.665097856 | 6.177547397 | 5.267410684 | 6.098063509 | 5.686130234 |
| NM_026386.1 | SNX2 | Mus musculus sorting nexin 2 (Snx2), mRNA. | 5.643115479 | 1.725093809 | 1.732280704 | 0.197442346 | 1.324947364 |
| NM_008198.1 | H2-BF | Mus musculus histocompatibility 2, complement component factor B (H2-Bf), mRNA. | 5.576813798 | 6.01291933 | 5.190685387 | 5.47998999 | 6.237024415 |
| XM_149096.1 | PRRX2 | Mus musculus paired related homeobox 2 (Prrx2), mRNA. | 5.5520945 | 5.730828931 | 5.210560624 | 3.956087515 | 0.744493646 |
| XM_149921.3 | WTIP |  | 5.496874304 | 4.044684664 | 4.900727844 | 1.475637011 | 1.095885699 |
| NM_011595.1 | TIMP3 | Mus musculus tissue inhibitor of metalloproteinase 3 (Timp3), mRNA. | 5.480673826 | 4.843389747 | 5.189379946 | 0.266856594 | 4.479809396 |
| NM_010171.2 | F3 | Mus musculus coagulation factor III (F3), mRNA. | 5.473574698 | 4.631690511 | 5.646581955 | 2.056124881 | 0.567943477 |
| NM_011413 | SLP | Mus musculus sex-limited protein (Slp), mRNA. | 5.446028189 | 5.647924999 | 5.78923569 | 6.542940188 | 6.535767058 |
| NM_013512.1 | EPB4.1L4A | Mus musculus erythrocyte protein band 4.1-like 4a (Epb4.1l4a), mRNA. | 5.44106247 | 5.676628209 | 5.048502056 | 4.212919361 | 4.679366368 |
| NM_013492.1 | CLU | Mus musculus clusterin (Clu), mRNA. | 5.440605839 | 1.577601512 | 4.973807336 | 6.956224079 | 4.323982255 |
| NM_012043.2 | ISLR | Mus musculus immunoglobulin superfamily containing leucine-rich repeat (Islr), mRNA. | 5.327232168 | 5.076089392 | 6.856010248 | 8.00308757 | 7.464424467 |
| XM_130954.3 | SLC27A3 | Mus musculus solute carrier family 27 (fatty acid transporter), member 3 (Slc27a3), mRNA. | 5.299861068 | 5.758490025 | 4.890474076 | 5.545801944 | 1.003207931 |
| NM_007969.2 | EXPI | Mus musculus extracellular proteinase inhibitor (Expi), mRNA. | 5.298304248 | 5.288815901 | 4.402033752 | 5.889735745 | 5.252701977 |
| NM_009750.1 | NGFRAP1 | Mus musculus nerve growth factor receptor (TNFRSF16) associated protein 1 (Ngfrap1), mRNA. | 5.238089353 | 4.189130313 | 5.141632982 | 0.765791905 | 0.247311658 |
| NM_008492.2 | LDH2 | Mus musculus lactate dehydrogenase 2, B chain (Ldh2), mRNA. | 5.228625346 | 4.120544831 | 5.213115266 | 5.825583701 | 1.871939766 |
| NM_009242 | SPARC | Mus musculus secreted acidic cysteine rich glycoprotein (Sparc), mRNA. | 5.221552434 | 4.382237838 | 5.754376732 | 0.933035284 | 3.782496586 |
| NM_133733.2 | 9030425E11RIK | Mus musculus RIKEN cDNA 9030425E11 gene (9030425E11Rik), mRNA. | 5.183882055 | 5.422349875 | 4.954306282 | 4.861536948 | 4.328972694 |
| NM_016903.2 | ESD |  | 5.137499069 | 1.7630904 | 1.915140032 | 0.616783863 | 0.382402423 |
| NM_172294.1 | SULF1 | Mus musculus sulfatase 1 (Sulf1), mRNA. | 5.055481885 | 5.084982683 | 4.558965439 | 1.596855169 | 0.385658094 |
| NM_033270.1 | E2F6 | Mus musculus E2F transcription factor 6 (E2f6), mRNA. | 5.044884731 | 4.363612308 | 4.18885548 | 1.023131195 | 2.322475471 |
| NM_178592.2 | BAT5 | Mus musculus HLA-B associated transcript 5 (Bat5), mRNA. | 5.005546351 | 5.168908117 | 3.965976936 | 4.648978557 | 0.322387359 |
| NM_153781.1 | PYGB | Mus musculus brain glycogen phosphorylase (Pygb), mRNA. | 5.004356521 | 3.725051586 | 4.462332679 | 5.017440647 | 2.749335841 |
| NM_009829.2 | CCND2 | Mus musculus cyclin D2 (Ccnd2), mRNA. | 4.970548603 | 5.783456915 | 6.415715727 | 4.221519117 | 5.525133186 |
| NM_009082.2 | RPL29 | Mus musculus ribosomal protein L29 (Rpl29), mRNA. | 4.960724547 | 5.680285093 | 4.51803275 | 5.844243833 | 5.727545557 |
| NM_011782.1 | ADAMTS5 | Mus musculus a disintegrin-like and metalloprotease (reprolysin type) with thrombospondin type 1 motif, 5 (aggrecanase-2) (Adamts5), mRNA. | 4.953818506 | 4.913512445 | 6.187622371 | 7.739908051 | 6.807001543 |
| NM_012011.1 | EIF2S3Y | Mus musculus eukaryotic translation initiation factor 2, subunit 3, structural gene Y-linked (Eif2s3y), mRNA. | 4.947777427 | 5.331349549 | 4.548971535 | 6.777404416 | 6.438336118 |
| NM_177757.3 | 4832420M10 | Mus musculus hypothetical protein 4832420M10 (4832420M10), mRNA. | 4.934418654 | 4.632126348 | 4.272256896 | 2.427788213 | 1.092131767 |
| NM_011311.1 | S100A4 | Mus musculus S100 calcium binding protein A4 (S100a4), mRNA. | 4.931171963 | 3.442872296 | 2.626361892 | 0.907950269 | 0.916403636 |
| NM_145711.2 | TOX | Mus musculus thymocyte selection-associated HMG box gene (Tox), mRNA. | 4.870687768 | 5.218363332 | 4.520025792 | 5.408626453 | 3.56461466 |
| NM_009054 | TRIM27 | Mus musculus tripartite motif protein 27 (Trim27), mRNA. | 4.860377014 | 0.885103484 | 1.1742673 | 1.83623544 | 2.387466617 |
| NM_008610.1 | MMP2 | Mus musculus matrix metalloproteinase 2 (Mmp2), mRNA. | 4.835146147 | 3.357502501 | 6.108824866 | 10.64992842 | 1.200675061 |
| NM_013864 | NDRG2 |  | 4.80231055 | 4.776962931 | 4.532419075 | 2.346721325 | 3.823875988 |
| NM_146068 | 2310008H04RIK | Mus musculus RIKEN cDNA 2310008H04 gene (2310008H04Rik), mRNA. | 4.798649461 | 1.972318982 | 1.861109188 | 3.908566685 | 3.609881851 |
| NM_010807.2 | MLP | Mus musculus MARCKS-like protein (Mlp), mRNA. | 4.780856517 | 5.217050623 | 4.421393618 | 2.876416933 | 1.162567252 |
| NM_173742.1 | D11BWG0434E | Mus musculus DNA segment, Chr 11, Brigham & Womens Genetics 0434 expressed (D11Bwg0434e), mRNA. | 4.772948241 | 0.680356532 | 1.106587204 | 0.809995177 | 1.723338414 |
| XM_128800.4 | MAP4K3 | Mus musculus mitogen-activated protein kinase kinase kinase kinase 3 (Map4k3), mRNA. | 4.76959645 | 5.005675606 | 4.863462154 | 5.307862479 | 4.247536609 |
| NM_134042.1 | ALDH6A1 | Mus musculus aldehyde dehydrogenase family 6, subfamily A1 (Aldh6a1), mRNA. | 4.760008879 | 3.939473079 | 5.358515247 | 3.431837073 | 4.942903826 |
| NM_177684.2 | ZFP637 |  | 4.749332851 | 4.483289064 | 3.890383874 | 3.592229722 | 3.216638255 |
| NM_007669.2 | CDKN1A | Mus musculus cyclin-dependent kinase inhibitor 1A (P21) (Cdkn1a), mRNA. | 4.695709299 | 2.10115538 | 2.764086371 | 1.576011255 | 6.556731124 |
| NM_007993 | FBN1 | Mus musculus fibrillin 1 (Fbn1), mRNA. | 4.693058739 | 3.77275815 | 5.800477432 | 0.667628198 | 0.085097959 |
| NM_016909.1 | TSNAX | Mus musculus translin-associated factor X (Tsnax), mRNA. | 4.666043784 | 4.655535586 | 4.450591932 | 0.987528151 | 2.376044668 |
| NM_011349.2 | SEMA3F | Mus musculus sema domain, immunoglobulin domain (Ig), short basic domain, secreted, (semaphorin) 3 F (Sema3f), mRNA. | 4.656612716 | 4.196921953 | 5.612367106 | 0.398057726 | 1.946351328 |
| XM_194370.3 | C130057K09 |  | 4.635981262 | 5.320961162 | 4.239432795 | 4.988725406 | 4.707589632 |
| NM_022417.1 | ITM2C | Mus musculus integral membrane protein 2C (Itm2c), mRNA. | 4.622154793 | 1.547770246 | 4.583564454 | 0.572999171 | 0.072814308 |
| NM_007802.2 | CTSK | Mus musculus cathepsin K (Ctsk), mRNA. | 4.617170843 | 3.703117339 | 3.841622516 | 0.939234703 | 4.529383303 |
| NM_007876.1 | DPEP1 | Mus musculus dipeptidase 1 (renal) (Dpep1), mRNA. | 4.585142301 | 3.961928072 | 5.023739599 | 6.79449441 | 5.601273373 |
| NM_009776 | SERPING1 | Mus musculus serine (or cysteine) proteinase inhibitor, clade G, member 1 (Serping1), mRNA. | 4.501941721 | 2.175700086 | 6.146395301 | 6.797535268 | 4.240805781 |
| NM_133187.1 | 1110032E23RIK | Mus musculus RIKEN cDNA 1110032E23 gene (1110032E23Rik), mRNA. | 4.495284871 | 4.912559822 | 4.924253519 | 0.435197229 | 1.224340915 |
| NM_025917.1 | 2010315L10RIK | Mus musculus RIKEN cDNA 2010315L10 gene (2010315L10Rik), mRNA. | 4.494166522 | 1.200524277 | 2.548353986 | 2.582434915 | 1.431920433 |
| XM_194109.2 | IGSF3 |  | 4.46334462 | 4.698387616 | 4.479155935 | 4.878831361 | 3.962899736 |
| NM_183171.1 | FEZ1 | Mus musculus fasciculation and elongation protein zeta 1 (zygin I) (Fez1), mRNA. | 4.451604977 | 4.303556404 | 4.311805775 | 2.153279011 | 1.77777269 |
| NM_138741.1 | SDPR | Mus musculus serum deprivation response (Sdpr), mRNA. | 4.431609753 | 3.892766398 | 4.472171779 | 0.627903403 | 3.868283601 |
| NM_183171.1 | FEZ1 | Mus musculus fasciculation and elongation protein zeta 1 (zygin I) (Fez1), mRNA. | 4.428320047 | 4.26340779 | 4.329912369 | 1.971468954 | 1.813770643 |
| NM_054041.1 | ANTXR1 | Mus musculus anthrax toxin receptor 1 (Antxr1), mRNA. | 4.416156909 | 3.760408619 | 4.952979518 | 1.845867279 | 0.988474105 |
| NM_130882.1 | CYP4F13 | Mus musculus cytochrome P450, family 4, subfamily f, polypeptide 13 (Cyp4f13), mRNA. | 4.408754767 | 2.596589868 | 4.090204724 | 3.000563578 | 2.84602305 |
| NM_148927.1 | PLEKHA4 | Mus musculus pleckstrin homology domain containing, family A (phosphoinositide binding specific) member 4 (Plekha4), mRNA. | 4.386211191 | 3.754806949 | 4.729775728 | 4.65556622 | 5.090787044 |
| NM_010360.1 | GSTM5 | Mus musculus glutathione S-transferase, mu 5 (Gstm5), mRNA. | 4.373098034 | 2.963857138 | 3.687565387 | 2.156695736 | 1.003375886 |
| NM_080456.1 | MRPS6 | Mus musculus mitochondrial ribosomal protein S6 (Mrps6), mRNA. | 4.359655014 | 6.116840029 | 4.852125871 | 5.513816659 | 5.565625086 |
| XM_358343.1 | SULF2 |  | 5.704314819 | 5.021212637 | 4.965757456 | 1.464283733 | 1.907561819 |
| NM_028472.1 | BMPER | Mus musculus BMP-binding endothelial regulator (Bmper), mRNA. | 4.358844545 | 5.24763723 | 4.248277046 | 4.481444413 | 4.458750256 |
| NM_145978.1 | PDLIM2 | Mus musculus PDZ and LIM domain 2 (Pdlim2), mRNA. | 4.357063315 | 4.189480099 | 4.20014976 | 5.026710583 | 5.171964439 |
| NM_025424.1 | 1110060M21RIK | Mus musculus RIKEN cDNA 1110060M21 gene (1110060M21Rik), mRNA. | 4.341133199 | 3.061159255 | 4.072643393 | 2.802984614 | 3.073048959 |
| NM_025807.1 | SLC16A9 | Mus musculus solute carrier family 16 (monocarboxylic acid transporters), member 9 (Slc16a9), mRNA. | 4.322343788 | 4.659400128 | 4.039443199 | 4.343493237 | 4.207416788 |
| NM_008088 | GAS7 | Mus musculus growth arrest specific 7 (Gas7), mRNA. | 4.318216222 | 4.192736739 | 3.948758572 | 2.880179177 | 2.150134147 |
| NM_028472.1 | BMPER | Mus musculus BMP-binding endothelial regulator (Bmper), mRNA. | 4.358844545 | 5.24763723 | 4.248277046 | 4.481444413 | 4.458750256 |
| NM_024244.3 | 1200015N20RIK | Mus musculus RIKEN cDNA 1200015N20 gene (1200015N20Rik), mRNA. | 4.28840762 | 4.748744927 | 3.857462375 | 2.764124887 | 3.672317545 |
| NM_019793.2 | TM4SF8 | Mus musculus transmembrane 4 superfamily member 8 (Tm4sf8), mRNA. | 4.281670208 | 5.150356156 | 4.692492534 | 2.822619388 | 4.879073453 |
| NM_009821.1 | RUNX1 | Mus musculus runt related transcription factor 1 (Runx1), mRNA. | 4.276222976 | 4.539379967 | 4.024017671 | 4.713770247 | 3.433308013 |
| NM_011338 | CCL9 | Mus musculus chemokine (C-C motif) ligand 9 (Ccl9), mRNA. | 4.233236799 | 4.342548609 | 5.416263324 | 11.13502993 | 11.00106664 |
| NM_024263 | 1200013A08RIK | Mus musculus RIKEN cDNA 1200013A08 gene (1200013A08Rik), mRNA. | 4.229279004 | 3.545517774 | 4.831668209 | 3.11675931 | 1.985617676 |
| XM_193573.3 | A230046K03RIK | Mus musculus RIKEN cDNA A230046K03 gene (A230046K03Rik), mRNA. | 4.214764956 | 1.971417366 | 1.508619411 | 1.362691474 | 1.996291047 |
| NM_029564 | TAX1BP3 |  | 4.210631619 | 3.757028467 | 3.538930923 | 1.452739234 | 1.300241715 |
| NM_011693.2 | VCAM1 | Mus musculus vascular cell adhesion molecule 1 (Vcam1), mRNA. | 4.206125468 | 2.751513778 | 4.863999314 | 3.104139136 | 3.836090552 |
| NM_009721.2 | ATP1B1 | Mus musculus ATPase, Na+/K+ transporting, beta 1 polypeptide (Atp1b1), mRNA. | 4.193704849 | 3.194035699 | 3.651180547 | 1.172694823 | 3.218273002 |
| NM_007669.2 | CDKN1A | Mus musculus cyclin-dependent kinase inhibitor 1A (P21) (Cdkn1a), mRNA. | 4.192615737 | 1.606295596 | 2.191943741 | 3.028838328 | 6.943236223 |
| NM_024244.3 | 1200015N20RIK | Mus musculus RIKEN cDNA 1200015N20 gene (1200015N20Rik), mRNA. | 4.28840762 | 4.748744927 | 3.857462375 | 2.764124887 | 3.672317545 |
| NM_011430.1 | SNCG | Mus musculus synuclein, gamma (Sncg), mRNA. | 4.152898842 | 4.829329863 | 3.850000669 | 5.081158146 | 2.583321104 |
| NM_027219.1 | CDC42EP1 | Mus musculus CDC42 effector protein (Rho GTPase binding) 1 (Cdc42ep1), mRNA. | 4.145140028 | 5.186662299 | 4.276367248 | 5.579768561 | 5.724718394 |
| NM_011695.1 | VDAC2 | Mus musculus voltage-dependent anion channel 2 (Vdac2), mRNA. | 4.144932317 | 1.942000292 | 1.441193332 | 2.508468655 | 2.667698472 |
| NM_183254.1 | 1700025K23RIK | Mus musculus RIKEN cDNA 1700025K23 gene (1700025K23Rik), mRNA. | 4.142339554 | 2.961011128 | 3.780679906 | 1.825024067 | 2.135806273 |
| NM_030888.2 | C1QTNF3 | Mus musculus C1q and tumor necrosis factor related protein 3 (C1qtnf3), mRNA. | 4.140574404 | 4.919821525 | 4.099984474 | 4.874114579 | 0.698385256 |
| NM_172607.2 | 9130210N20RIK | Mus musculus RIKEN cDNA 9130210N20 gene (9130210N20Rik), mRNA. | 4.122953202 | 2.842053207 | 3.744114332 | 1.036418251 | 1.045467225 |
| NM_025830.3 | 1300010O06RIK | 4.102476924 | 2.330289541 | 1.285247649 | 1.340289998 | 1.658896134 | NM_025830.3 |
| NM_145978.1 | PDLIM2 | Mus musculus PDZ and LIM domain 2 (Pdlim2), mRNA. | 4.075303074 | 4.15229085 | 3.696240261 | 4.049227979 | 4.411884966 |
| NM_178939.1 | PDRG1 |  | 4.056041892 | 3.156052896 | 3.66642809 | 0.642030506 | 4.001859956 |
| NM_013494.2 | CPE | Mus musculus carboxypeptidase E (Cpe), mRNA. | 4.054780184 | 2.686405968 | 5.37923589 | 1.481540762 | 4.689516756 |
| NM_027727.1 | 4933427L07RIK | Mus musculus RIKEN cDNA 4933427L07 gene (4933427L07Rik), mRNA. | 4.047218728 | 0.949471691 | 0.166553492 | 2.100027771 | 1.252735134 |
| NM_020595 | OTOR | Mus musculus otoraplin (Otor), mRNA. | 4.039070352 | 4.375165714 | 3.813314638 | 4.607143965 | 4.573536082 |
| NM_053078.3 | D0H4S114 | Mus musculus DNA segment, human D4S114 (D0H4S114), mRNA. | 4.026449425 | 3.345917057 | 3.576560103 | 0.319569259 | 1.281572194 |
| NM_025711.2 | ASPN | Mus musculus asporin (Aspn), mRNA. | 4.015490881 | 4.360792204 | 3.554450493 | 4.487121889 | 2.056934371 |
| NM_018827.1 | CRLF1 | Mus musculus cytokine receptor-like factor 1 (Crlf1), mRNA. | 4.003887839 | 4.452898222 | 4.673266982 | 6.109993478 | 2.934357279 |
| NM_028810.1 | RHOE | Mus musculus ras homolog gene family, member E (Rhoe), mRNA. | 3.991970618 | 1.443934477 | 2.061332988 | 2.630076229 | 1.591556364 |
| NM_183251.2 | 1810020D17RIK | Mus musculus RIKEN cDNA 1810020D17 gene (1810020D17Rik), mRNA. | 3.987567263 | 1.489131533 | 2.067309503 | 0.822987638 | 1.57757368 |
| NM_017370.1 | HP | Mus musculus haptoglobin (Hp), mRNA. | 3.97888359 | 0.012071305 | 11.06723346 | 14.41285999 | 13.25354515 |
| NM_144896.2 | PET112L | Mus musculus PET112-like (yeast) (Pet112l), mRNA. | 3.933291021 | 4.620021622 | 3.636224326 | 4.355035907 | 4.31941219 |
| NM_054041.1 | ANTXR1 | Mus musculus anthrax toxin receptor 1 (Antxr1), mRNA. | 3.932260964 | 3.136705297 | 4.054802941 | 1.463862256 | 0.781143356 |
| XM_359276.1 | D930038M13RIK | Mus musculus RIKEN cDNA D930038M13 gene (D930038M13Rik), mRNA. | 3.889424361 | 3.689241331 | 3.584825117 | 2.53289011 | 2.822774843 |
| NM_026931.1 | 1810011O10RIK | Mus musculus RIKEN cDNA 1810011O10 gene (1810011O10Rik), mRNA. | 3.870285942 | 4.053906642 | 3.580859762 | 3.975735767 | 3.700267904 |
| NM_027309.1 | 2210402C18RIK | Mus musculus RIKEN cDNA 2210402C18 gene (2210402C18Rik), mRNA. | 3.869835824 | 3.8143942 | 3.744622137 | 1.814482362 | 2.765360508 |
| NM_207264.2 | BC052040 | Mus musculus cDNA sequence BC052040 (BC052040), mRNA. | 3.862753384 | 0.069474645 | 0.123295924 | 0.387795805 | 1.54543747 |
| NM_172865.2 | MANEA |  | 3.811637604 | 4.332874147 | 4.186117404 | 3.35306056 | 4.127041844 |
| NM_019479.2 | HES6 | Mus musculus hairy and enhancer of split 6 (Drosophila) (Hes6), mRNA. | 3.804396369 | 1.66761724 | 2.871777277 | 1.890291264 | 0.402895398 |
| NM_029803.1 | 2310061N23RIK | Mus musculus RIKEN cDNA 2310061N23 gene (2310061N23Rik), mRNA. | 3.786725865 | 0.147270835 | 4.756373549 | 4.866396071 | 1.739351946 |
| NM_008987.2 | PTX3 | Mus musculus pentaxin related gene (Ptx3), mRNA. | 3.773862764 | 6.099652065 | 5.757439959 | 10.29478378 | 4.759324761 |
| XM_125538.4 | SESN1 | Mus musculus sestrin 1 (Sesn1), mRNA. | 3.771924075 | 3.037569252 | 3.69957671 | 1.670254825 | 3.891272372 |
| NM_008064.2 | GAA | Mus musculus glucosidase, alpha, acid (Gaa), mRNA. | 3.768490653 | 1.632594359 | 3.636439005 | 1.110240163 | 0.14387292 |
| NM_181404.3 | D330024H06RIK | Mus musculus RIKEN cDNA D330024H06 gene (D330024H06Rik), mRNA. | 3.76551957 | 3.743707175 | 3.646392855 | 1.943354227 | 3.299991116 |
| NM_172525.1 | B130017I01RIK | Mus musculus RIKEN cDNA B130017I01 gene (B130017I01Rik), mRNA. | 3.763118146 | 4.144072613 | 4.237102406 | 1.640513421 | 2.346006646 |
| XM_140742.4 | 1110018J23RIK | Mus musculus RIKEN cDNA 1110018J23 gene (1110018J23Rik), mRNA. | 3.750858512 | 2.251426967 | 1.965365906 | 2.36383229 | 2.286266741 |
| NM_029803.1 | 2310061N23RIK | Mus musculus RIKEN cDNA 2310061N23 gene (2310061N23Rik), mRNA. | 3.786725865 | 0.147270835 | 4.756373549 | 4.866396071 | 1.739351946 |
| NM_024477.2 | AI428795 | Mus musculus expressed sequence AI428795 (AI428795), mRNA. | 3.748945254 | 1.092708106 | 2.203349731 | 1.703774058 | 1.007347914 |
| NM_033617.1 | ATP6V0B | Mus musculus ATPase, H+ transporting, V0 subunit B (Atp6v0b), mRNA. | 3.748804213 | 2.000886781 | 2.509021614 | 0.134518336 | 0.646038439 |
| NM_007806.1 | CYBA | Mus musculus cytochrome b-245, alpha polypeptide (Cyba), mRNA. | 3.748740801 | 1.481022461 | 2.822294621 | 0.675137166 | 1.957698619 |
| NM_011563.2 | PRDX2 | Mus musculus peroxiredoxin 2 (Prdx2), mRNA. | 3.746309083 | 4.361703826 | 3.882420151 | 4.581622157 | 4.515696649 |
| XM_147935.1 | PRICKLE1 | Mus musculus prickle like 1 (Drosophila) (Prickle1), mRNA. | 3.726146901 | 4.150394696 | 4.267097219 | 1.38737342 | 0.244629267 |
| NM_010825 | MRG1 | Mus musculus myeloid ecotropic viral integration site-related gene 1 (Mrg1), mRNA. | 3.704515815 | 4.690609485 | 4.656024095 | 5.156347878 | 5.614838122 |
| NM_026428.1 | DCXR | Mus musculus dicarbonyl L-xylulose reductase (Dcxr), mRNA. | 3.698543905 | 1.85730472 | 3.253683258 | 1.834818309 | 2.37116218 |
| NM_024495.2 | CAR13 | Mus musculus carbonic anhydrase 13 (Car13), mRNA. | 3.694246233 | 3.691611939 | 2.666223974 | 4.88894777 | 4.767493548 |
| NM_025427.1 | 1190002H23RIK | Mus musculus RIKEN cDNA 1190002H23 gene (1190002H23Rik), mRNA. | 3.688936467 | 3.657098724 | 2.996144623 | 3.892130206 | 1.153891728 |
| NM_134189.2 | GALNT10 | Mus musculus UDP-N-acetyl-alpha-D-galactosamine:polypeptide N-acetylgalactosaminyltransferase 10 (Galnt10), mRNA. | 3.665976768 | 3.866891411 | 2.92628769 | 1.278820876 | 0.252827731 |
| NM_008183.2 | GSTM2 | Mus musculus glutathione S-transferase, mu 2 (Gstm2), mRNA. | 3.663814101 | 1.356258515 | 5.642609633 | 2.481874679 | 3.094475707 |
| NM_026405.2 | RAB32 | Mus musculus RAB32, member RAS oncogene family (Rab32), mRNA. | 3.654414925 | 4.889793643 | 3.604014901 | 6.075017051 | 8.501725511 |
| NM_026428.1 | DCXR | Mus musculus dicarbonyl L-xylulose reductase (Dcxr), mRNA. | 3.698543905 | 1.85730472 | 3.253683258 | 1.834818309 | 2.37116218 |
| NM_024495.2 | CAR13 | Mus musculus carbonic anhydrase 13 (Car13), mRNA. | 3.694246233 | 3.691611939 | 2.666223974 | 4.88894777 | 4.767493548 |
| XM_125970.2 | 2410008K03RIK | Mus musculus RIKEN cDNA 2410008K03 gene (2410008K03Rik), mRNA. | 3.638122644 | 3.61266521 | 3.530644067 | 0.843639659 | 0.926878688 |
| NM_029564 | TAX1BP3 |  | 3.631290814 | 3.915328659 | 3.167665078 | 1.620439987 | 1.247171894 |
| NM_025428.1 | ZDHHC12 | Mus musculus zinc finger, DHHC domain containing 12 (Zdhhc12), mRNA. | 3.62436931 | 0.554877048 | 1.358117278 | 2.417211265 | 2.145200295 |
| NM_026993.1 | DDAH1 | Mus musculus dimethylarginine dimethylaminohydrolase 1 (Ddah1), mRNA. | 3.624143182 | 2.556707869 | 2.987592255 | 2.16697389 | 2.095132942 |
| XM_283793.2 | MYL9 | Mus musculus myosin, light polypeptide 9, regulatory (Myl9), mRNA. | 3.61991516 | 2.121387554 | 3.347848407 | 0.064990388 | 1.993416489 |
| NM_026821.2 | D4BWG0951E | Mus musculus DNA segment, Chr 4, Brigham & Womens Genetics 0951 expressed (D4Bwg0951e), mRNA. | 3.576425245 | 3.338606448 | 2.459127192 | 1.706065132 | 0.561148806 |
| NM_019427 | EPB4.1L4B | Mus musculus erythrocyte protein band 4.1-like 4b (Epb4.1l4b), mRNA. | 3.568090202 | 3.580863661 | 3.231068063 | 4.145333331 | 3.540283691 |
| NM_021715.1 | CHST7 | Mus musculus carbohydrate (N-acetylglucosamino) sulfotransferase 7 (Chst7), mRNA. | 3.565558336 | 3.777494709 | 3.277906283 | 3.694118602 | 2.956795466 |
| NM_008687.2 | NFIB | Mus musculus nuclear factor I/B (Nfib), mRNA. | 3.564991244 | 4.67090716 | 4.780006677 | 4.487124971 | 5.662686856 |
| NM_007564.2 | ZFP36L1 | Mus musculus zinc finger protein 36, C3H type-like 1 (Zfp36l1), mRNA. | 3.559470171 | 2.11737021 | 5.796387679 | 3.804678255 | 3.960835126 |
| XM_125970.2 | 2410008K03RIK | Mus musculus RIKEN cDNA 2410008K03 gene (2410008K03Rik), mRNA. | 3.638122644 | 3.61266521 | 3.530644067 | 0.843639659 | 0.926878688 |
| NM_029564 | TAX1BP3 |  | 3.631290814 | 3.915328659 | 3.167665078 | 1.620439987 | 1.247171894 |
| NM_054071.1 | FGFRL1 | Mus musculus fibroblast growth factor receptor-like 1 (Fgfrl1), mRNA. | 3.545409041 | 3.499242105 | 3.544855347 | 5.755176729 | 5.104354363 |
| XM_131700.4 | BC039093 | Mus musculus cDNA sequence BC039093 (BC039093), mRNA. | 3.535393914 | 3.710477861 | 3.177457271 | 1.862277017 | 1.353033444 |
| NM_010884.1 | NDRG1 |  | 3.509138275 | 0.951234924 | 3.294383825 | 0.029061576 | 2.299427723 |
| NM_008885.1 | PMP22 | Mus musculus peripheral myelin protein (Pmp22), mRNA. | 3.492744994 | 2.939274446 | 3.543087 | 2.296649641 | 1.538602463 |
| NM_029565.2 | ORF18 | Mus musculus open reading frame 18 (ORF18), mRNA. | 3.49043806 | 2.863405169 | 3.655473393 | 0.888650075 | 0.791237798 |
| NM_010430.1 | HIC1 | Mus musculus hypermethylated in cancer 1 (Hic1), mRNA. | 3.488196808 | 3.165050564 | 3.32318251 | 0.475879824 | 0.339084094 |
| NM_054071.1 | FGFRL1 | Mus musculus fibroblast growth factor receptor-like 1 (Fgfrl1), mRNA. | 3.545409041 | 3.499242105 | 3.544855347 | 5.755176729 | 5.104354363 |
| NM_013750.1 | PHLDA3 | Mus musculus pleckstrin homology-like domain, family A, member 3 (Phlda3), mRNA. | 3.479203126 | 3.653524456 | 2.248064687 | 2.20989034 | 0.239586071 |
| NM_008183.2 | GSTM2 | Mus musculus glutathione S-transferase, mu 2 (Gstm2), mRNA. | 3.464441706 | 1.587815078 | 4.490929441 | 1.473032356 | 2.77183026 |
| NM_172393.1 | AIM1 | Mus musculus absent in melanoma 1 (Aim1), mRNA. | 3.444414695 | 3.431876938 | 3.066746527 | 3.55338524 | 3.722552527 |
| NM_011356.2 | FRZB | Mus musculus frizzled-related protein (Frzb), mRNA. | 3.442158771 | 3.720402885 | 3.29145736 | 2.555232336 | 3.592145883 |
| NM_028608.1 | GLIPR1 | Mus musculus GLI pathogenesis-related 1 (glioma) (Glipr1), mRNA. | 3.434935017 | 2.979545752 | 2.22686109 | 6.140552961 | 7.315177786 |
| NM_028027.1 | D10ERTD610E | Mus musculus DNA segment, Chr 10, ERATO Doi 610, expressed (D10Ertd610e), mRNA. | 3.433615681 | 1.977697981 | 2.91958235 | 3.102540611 | 1.655847963 |
| XM_128555.4 | EME2 |  | 3.42745972 | 3.471895522 | 2.790508716 | 3.549900294 | 3.573940298 |
| NM_010258.2 | GATA6 | Mus musculus GATA binding protein 6 (Gata6), mRNA. | 3.422682765 | 2.437440216 | 3.260414142 | 0.773002441 | 3.823262986 |
| XM_283793.2 | MYL9 | Mus musculus myosin, light polypeptide 9, regulatory (Myl9), mRNA. | 3.405986134 | 2.18346605 | 2.962815653 | 0.413030654 | 1.912809661 |
| NM_019953.1 | TMEM4 | Mus musculus transmembrane protein 4 (Tmem4), mRNA. | 3.381521893 | 3.034098966 | 2.678355454 | 2.325168983 | 2.343402593 |
| NM_021897.1 | TRP53INP1 | Mus musculus transformation related protein 53 inducible nuclear protein 1 (Trp53inp1), mRNA. | 3.37486412 | 3.437044996 | 3.204582477 | 1.976312354 | 0.876223462 |
| NM_080555.1 | PPAP2B | Mus musculus phosphatidic acid phosphatase type 2B (Ppap2b), mRNA. | 3.372872278 | 2.395797626 | 3.70845653 | 7.357493805 | 5.217962733 |
| NM_010156.2 | AA175286 | Mus musculus EST AA175286 (AA175286), mRNA. | 3.364635911 | 3.522086908 | 3.385582488 | 1.228953941 | 1.91500489 |
| NM_011905.2 | TLR2 | Mus musculus toll-like receptor 2 (Tlr2), mRNA. | 3.363377623 | 2.884205837 | 3.841258887 | 3.388891992 | 4.21454815 |
| NM_028142.1 | 2810405F18RIK | Mus musculus RIKEN cDNA 2810405F18 gene (2810405F18Rik), mRNA. | 3.363004746 | 3.59219084 | 2.994733364 | 2.69074348 | 2.611501621 |
| NM_009169.1 | SHFDG1 | Mus musculus split hand/foot deleted gene 1 (Shfdg1), mRNA. | 3.358420984 | 3.674138832 | 3.234344561 | 2.79645605 | 2.744383106 |
| NM_028044.1 | CNN3 | Mus musculus calponin 3, acidic (Cnn3), mRNA. | 3.355724275 | 2.615555324 | 3.422398343 | 2.103893692 | 1.722151893 |
| NM_024427 | TPM1 | Mus musculus tropomyosin 1, alpha (Tpm1), mRNA. | 3.349764891 | 3.171406293 | 3.399342975 | 1.733537374 | 3.905443841 |
| NM_178660.2 | RBMS3 |  | 3.342336111 | 3.076735782 | 4.0647911 | 1.00947779 | 1.887951819 |
| NM_133903.2 | SPON2 | Mus musculus spondin 2, extracellular matrix protein (Spon2), mRNA. | 3.337010442 | 2.266502778 | 6.921929559 | 1.735815986 | 4.579505719 |
| NM_008452.1 | KLF2 | Mus musculus Kruppel-like factor 2 (lung) (Klf2), mRNA. | 3.335396166 | 1.619957416 | 2.520864628 | 1.708735131 | 2.826244288 |
| NM_139198.1 | PLAC8 | Mus musculus placenta-specific 8 (Plac8), mRNA. | 3.323867585 | 1.532344027 | 4.224968269 | 0.753613918 | 1.764399096 |
| NM_011594.2 | TIMP2 | Mus musculus tissue inhibitor of metalloproteinase 2 (Timp2), mRNA. | 3.318622613 | 2.072585731 | 4.19887155 | 1.66333628 | 1.851377916 |
| NM_023055.1 | SLC9A3R2 | Mus musculus solute carrier family 9 (sodium/hydrogen exchanger), isoform 3 regulator 2 (Slc9a3r2), mRNA. | 3.315529693 | 3.092601126 | 2.974729368 | 0.957519617 | 0.15222679 |
| NM_019656 | TM4SF6 | Mus musculus transmembrane 4 superfamily member 6 (Tm4sf6), mRNA. | 3.305866224 | 3.26627968 | 3.789783765 | 3.130639936 | 3.133797132 |
| XM_128764.3 | VIT | Mus musculus vitrin (Vit), mRNA. | 3.300745537 | 2.967468683 | 3.138798503 | 0.145193193 | 4.303574181 |
| XM_128594.4 | NDUFB10 | Mus musculus NADH dehydrogenase (ubiquinone) 1 beta subcomplex, 10 (Ndufb10), mRNA. | 3.298611575 | 3.369548548 | 1.871578509 | 3.82939341 | 3.235457312 |
| NM_145220.1 | DIP3B | Mus musculus Dip3 beta (Dip3b), mRNA. | 3.295390619 | 3.044607732 | 3.151083795 | 2.055702897 | 2.938537909 |
| NM_008538 | MARCKS | Mus musculus myristoylated alanine rich protein kinase C substrate (Marcks), mRNA. | 3.294801027 | 3.967937037 | 5.627165714 | 4.93759716 | 3.293080916 |
| NM_030700.1 | MAGED2 | Mus musculus melanoma antigen, family D, 2 (Maged2), mRNA. | 3.293507411 | 2.490417231 | 4.839716324 | 7.285457635 | 3.820947979 |
| NM_146194 | PICALM | Mus musculus phosphatidylinositol binding clathrin assembly protein (Picalm), mRNA. | 3.276978165 | 3.563530518 | 3.39344966 | 3.275451775 | 2.909289659 |
| NM_023670.2 | IGF2BP3 | Mus musculus insulin-like growth factor 2, binding protein 3 (Igf2bp3), mRNA. | 3.272003724 | 3.963984439 | 3.520909443 | 4.262073149 | 1.086857532 |
| NM_009713.1 | ARSA | Mus musculus arylsulfatase A (Arsa), mRNA. | 3.261875732 | 1.189561331 | 3.163908566 | 0.382487578 | 0.578104357 |
| XM_109683.4 | 1810027O10RIK | Mus musculus RIKEN cDNA 1810027O10 gene (1810027O10Rik), mRNA. | 3.261167573 | 2.4146562 | 2.821009151 | 2.393015336 | 1.987604228 |
| NM_016873.1 | WISP2 | Mus musculus WNT1 inducible signaling pathway protein 2 (Wisp2), mRNA. | 3.259207402 | 0.1456542 | 5.65913351 | 2.928070644 | 2.484190403 |
| NM_013685.1 | TCF4 | Mus musculus transcription factor 4 (Tcf4), mRNA. | 3.255652684 | 3.645221834 | 4.856196811 | 0.480148713 | 3.568223743 |
| NM_023670.2 | IGF2BP3 | Mus musculus insulin-like growth factor 2, binding protein 3 (Igf2bp3), mRNA. | 3.246589594 | 3.914869115 | 3.104381427 | 3.995368001 | 0.973968175 |
| NM_181585.3 | PIK3R3 | Mus musculus phosphatidylinositol 3 kinase, regulatory subunit, polypeptide 3 (p55) (Pik3r3), mRNA. | 3.24470847 | 3.680345899 | 3.173083441 | 2.187427153 | 2.409440177 |
| NM_172958.2 | PIP3AP | Mus musculus phosphatidylinositol-3-phosphatase associated protein (Pip3ap), mRNA. | 3.240699248 | 0.522321271 | 0.315248384 | 0.319301774 | 1.694874577 |
| NM_029823.1 | 6530401D17RIK | Mus musculus RIKEN cDNA 6530401D17 gene (6530401D17Rik), mRNA. | 3.232171846 | 2.698030142 | 2.734384975 | 2.07435296 | 0.167875562 |
| NM_007825.1 | CYP7B1 | Mus musculus cytochrome P450, family 7, subfamily b, polypeptide 1 (Cyp7b1), mRNA. | 3.229957625 | 2.29054329 | 3.087291367 | 2.167214135 | 0.505827017 |
| NM_010299.2 | GM2A | Mus musculus GM2 ganglioside activator protein (Gm2a), mRNA. | 3.225844693 | 2.267207922 | 2.968707034 | 2.496681247 | 0.264368645 |
| NM_011023.2 | OTX1 | Mus musculus orthodenticle homolog 1 (Drosophila) (Otx1), mRNA. | 3.224548802 | 3.5311291 | 3.017665919 | 2.044774201 | 3.642454771 |
| NM_033509.2 | LTAP | Mus musculus loop tail associated protein (Ltap), mRNA. | 3.206890777 | 3.082523727 | 3.046487734 | 2.175701136 | 1.841465474 |
| NM_021454.3 | CDC42EP5 | Mus musculus CDC42 effector protein (Rho GTPase binding) 5 (Cdc42ep5), mRNA. | 3.205458479 | 0.503142741 | 0.207542584 | 0.966217375 | 1.870010559 |
| NM_010753.2 | MXD4 |  | 3.20030287 | 2.91520288 | 3.507208478 | 3.794479614 | 2.020749111 |
| NM_029406.1 | 1110061L23RIK | Mus musculus RIKEN cDNA 1110061L23 gene (1110061L23Rik), mRNA. | 3.197025976 | 0.36770618 | 0.684211463 | 0.724728946 | 1.585556523 |
| NM_008750.2 | NXN | Mus musculus nucleoredoxin (Nxn), mRNA. | 3.188692055 | 2.265835719 | 2.02522689 | 2.24085984 | 1.40128691 |
| NM_007899 | ECM1 | Mus musculus extracellular matrix protein 1 (Ecm1), mRNA. | 3.18571619 | 0.115976839 | 1.542730078 | 1.300746986 | 0.211952356 |
| NM_026158.1 | 0610042E07RIK | Mus musculus RIKEN cDNA 0610042E07 gene (0610042E07Rik), mRNA. | 3.175791363 | 2.740662519 | 3.139674108 | 3.554109855 | 3.00852814 |
| NM_021604.2 | AGRN | Mus musculus agrin (Agrn), mRNA. | 3.172429396 | 0.34149868 | 3.742709608 | 3.132801008 | 0.722852798 |
| NM_022814.1 | POLYDOM | Mus musculus polydomain protein (Polydom), mRNA. | 3.172258471 | 2.746759427 | 2.847910075 | 2.427839917 | 0.416375475 |
| NM_145978.1 | PDLIM2 | Mus musculus PDZ and LIM domain 2 (Pdlim2), mRNA. | 3.16881936 | 3.249224515 | 3.08971577 | 3.276549831 | 3.922981863 |
| NM_146067 | C530044N13RIK | Mus musculus RIKEN cDNA C530044N13 gene (C530044N13Rik), mRNA. | 3.166965208 | 3.529692393 | 2.671851938 | 1.122904802 | 0.867572468 |
| NM_031166.1 | IDB4 | Mus musculus inhibitor of DNA binding 4 (Idb4), mRNA. | 3.163357674 | 2.807036191 | 2.67289194 | 2.572435195 | 1.788063343 |
| XM_358863.1 | CBLB | Mus musculus Casitas B-lineage lymphoma b (Cblb), mRNA. | 3.161036763 | 0.205557203 | 1.356738819 | 1.289700298 | 0.036351578 |
| NM_009144.1 | SFRP2 | Mus musculus secreted frizzled-related sequence protein 2 (Sfrp2), mRNA. | 3.158755978 | 3.486913067 | 2.754034017 | 3.09127489 | 2.573649121 |
| NM_172648 | IFI205 | Mus musculus interferon activated gene 205 (Ifi205), mRNA. | 3.154801603 | 3.200347301 | 3.051018542 | 3.781300166 | 3.95760297 |
| NM_178884.2 | AW822216 | Mus musculus expressed sequence AW822216 (AW822216), mRNA. | 3.1511815 | 2.663771307 | 2.558089151 | 0.094534101 | 1.522428718 |
| NM_175260.1 | MYH10 | Mus musculus myosin heavy chain 10, non-muscle (Myh10), mRNA. | 3.146129315 | 3.731363918 | 3.52437303 | 2.903605008 | 2.61364203 |
| NM_010764 | MAN2B1 | Mus musculus mannosidase 2, alpha B1 (Man2b1), mRNA. | 3.138409265 | 2.428658043 | 3.028584226 | 2.58184031 | 0.359792305 |
| NM_025436.1 | SC4MOL | Mus musculus sterol-C4-methyl oxidase-like (Sc4mol), mRNA. | 3.130244914 | 3.337297756 | 2.916511158 | 0.333161552 | 0.369971249 |
| NM_008608.2 | MMP14 | Mus musculus matrix metalloproteinase 14 (membrane-inserted) (Mmp14), mRNA. | 3.121763137 | 3.014070853 | 2.788047173 | 0.227495314 | 2.668120039 |
| NM_172296.1 | DMRTA2 | Mus musculus doublesex and mab-3 related transcription factor like family A2 (Dmrta2), mRNA. | 3.12157441 | 3.241678355 | 3.355251779 | 1.043283485 | 3.329426797 |
| NM_153790.1 | SCARF2 | Mus musculus scavenger receptor class F, member 2 (Scarf2), mRNA. | 3.109444214 | 2.804201409 | 2.244315904 | 2.273122117 | 0.869196306 |
| NM_023699.2 | NFATC4 | Mus musculus nuclear factor of activated T-cells, cytoplasmic, calcineurin-dependent 4 (Nfatc4), mRNA. | 3.090096169 | 2.975988329 | 3.240958565 | 2.688122097 | 2.827573939 |
| NM_138721.1 | LSM10 | Mus musculus U7 snRNP-specific Sm-like protein LSM10 (Lsm10), mRNA. | 3.088657062 | 2.601595721 | 2.279121563 | 1.068475093 | 0.728194587 |
| NM_019427 | EPB4.1L4B | Mus musculus erythrocyte protein band 4.1-like 4b (Epb4.1l4b), mRNA. | 3.086628722 | 3.263586965 | 2.796290929 | 3.441475667 | 2.974994941 |
| NM_133986.1 | TCTA |  | 3.081921075 | 0.552677781 | 3.254354611 | 0.711876092 | 0.465794155 |
| NM_033616.2 | CSPRS | Mus musculus component of Sp100-rs (Csprs), mRNA. | 3.072158816 | 1.770305547 | 3.71619766 | 2.184583535 | 4.59852301 |
| NM_024462.1 | 2410005K17RIK | Mus musculus RIKEN cDNA 2410005K17 gene (2410005K17Rik), mRNA. | 3.056276152 | 4.052981338 | 3.443860212 | 3.330381816 | 2.871586097 |
| NM_033370 | COPB1 | Mus musculus coatomer protein complex, subunit beta 1 (Copb1), mRNA. | 3.054577347 | 2.912385363 | 2.638546466 | 0.607851166 | 0.595452095 |
| NM_025390.2 | POP4 | Mus musculus processing of precursor 4, ribonuclease P/MRP family, (S. cerevisiae) (Pop4), mRNA. | 3.045042767 | 3.763631792 | 2.403007398 | 3.87290668 | 4.109249508 |
| NM_009255.2 | SERPINE2 | Mus musculus serine (or cysteine) proteinase inhibitor, clade E, member 2 (Serpine2), mRNA. | 3.043492602 | 3.419083693 | 3.049540569 | 3.418067864 | 0.012340246 |
| NM_024217.2 | CKLFSF3 | Mus musculus chemokine-like factor super family 3 (Cklfsf3), mRNA. | 3.043274227 | 1.931001516 | 2.576452834 | 0.466605891 | 1.682820856 |
| NM_013587.1 | LRPAP1 | Mus musculus low density lipoprotein receptor-related protein associated protein 1 (Lrpap1), mRNA. | 3.033920115 | 2.06231496 | 2.872061654 | 1.81029735 | 2.232432586 |
| NM_020026.2 | B3GALT3 | Mus musculus UDP-Gal:betaGlcNAc beta 1,3-galactosyltransferase, polypeptide 3 (B3galt3), mRNA. | 3.028225086 | 3.48526828 | 3.197261231 | 2.155937369 | 1.479985494 |
| NM_010906.1 | NFIX | Mus musculus nuclear factor I/X (Nfix), mRNA. | 3.027508061 | 4.113670833 | 4.282897747 | 3.487992127 | 2.677641566 |
| NM_008714.2 | NOTCH1 | Mus musculus Notch gene homolog 1 (Drosophila) (Notch1), mRNA. | 3.016597161 | 3.22490882 | 3.827321478 | 3.987936475 | 3.243972019 |
| NM_009503.2 | VCP | Mus musculus valosin containing protein (Vcp), mRNA. | 3.012638223 | 4.529110516 | 2.364778825 | 2.917761349 | 1.643537236 |
| XM_131537.4 | 6430559E15RIK | Mus musculus RIKEN cDNA 6430559E15 gene (6430559E15Rik), mRNA. | 3.010710513 | 0.562660383 | 2.396854944 | 3.486710515 | 1.514028752 |
| NM_030690.2 | RAI14 | Mus musculus retinoic acid induced 14 (Rai14), mRNA. | 3.008264439 | 3.38301298 | 3.604975859 | 4.051932535 | 4.691906979 |
| NM_009177.2 | SIAT4A | Mus musculus sialyltransferase 4A (beta-galactoside alpha-2,3-sialytransferase) (Siat4a), mRNA. | 3.001181087 | 1.280939138 | 1.942304803 | 1.493167767 | 0.30178777 |

The 288 genes highly expressed in PA6-DA cells as compared to the PA6-X cell subtype (Z-ratio ≥ 3.0). The table shows relative expression of these genes in PA6-DA cells as compared to the transformed stromal cell lines, PA6-X1 and MS5, and to the MM55K and MEF cells, as Z-ratios.
